# Supplementary material for: SUMO-Chain-Regulated Proteasomal Degradation Timing Exemplified in DNA Replication Initiation
Source: Mol Cell. 2019 Nov 21;76(4):632–645.e6. doi: 10.1016/j.molcel.2019.08.003 (PMC6891891; doi:10.1016/j.molcel.2019.08.003)
Supplement: Document S1. Figures S1–S7 [file mmc1.pdf]

Molecular Cell, Volume *76*

Supplemental Information

SUMO-Chain-Regulated Proteasomal Degradation Timing Exemplified in DNA Replication Initiation

Ivan Psakhye, Federica Castellucci, and Dana Branzei

Figure S1

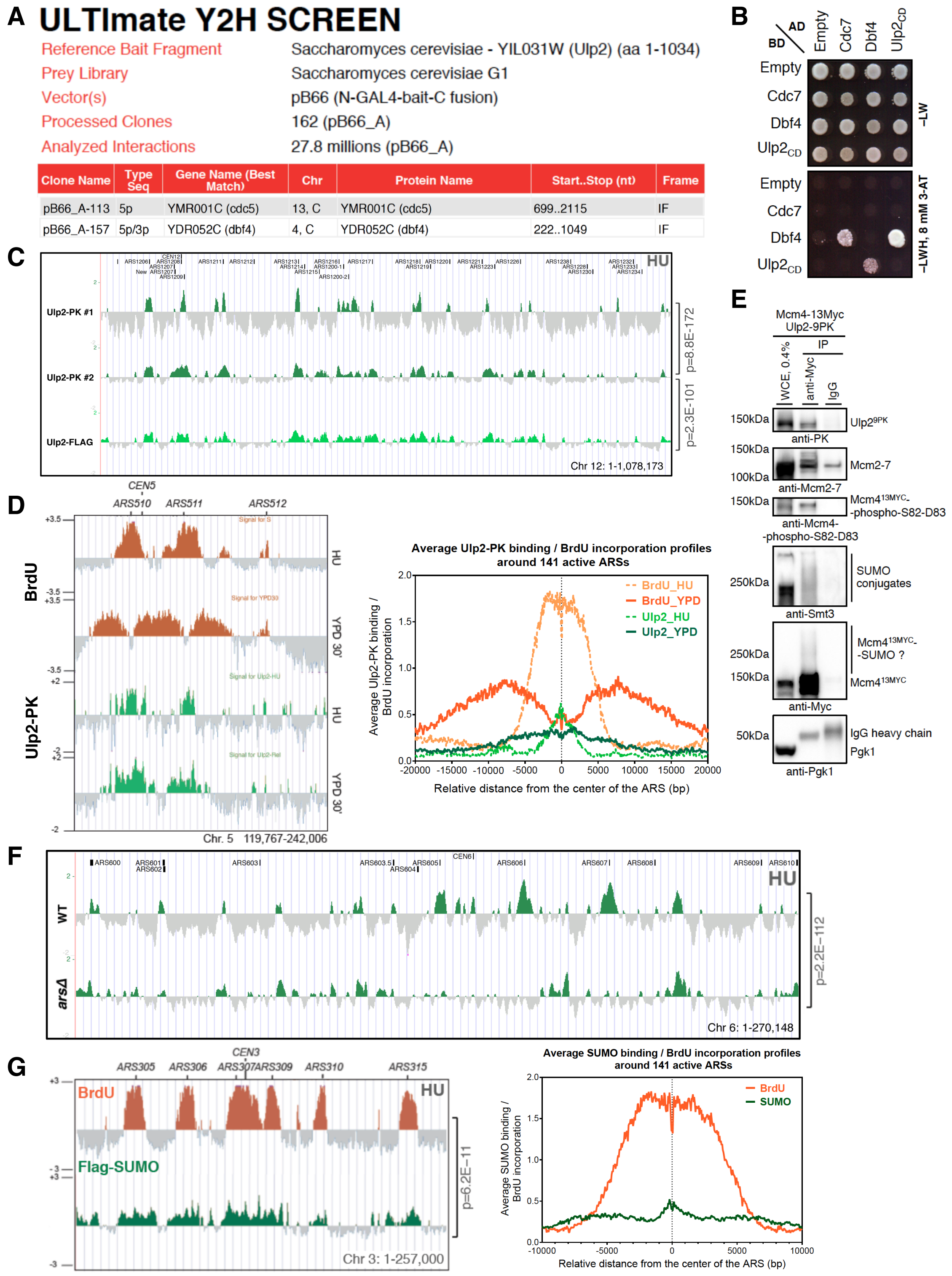

Figure S1. SUMO-deconjugating Enzyme Ulp2 Interacts with DDK and Is Recruited to Replication Origins, Related to Figure 1.

(A) The parameters of the ULTImate Y2H screen performed by Hybrigenics using full-length Ulp2 as bait. The fragments of the Polo-like kinase Cdc5 and the regulatory subunit of DDK, Dbf4, identified as interactors of Ulp2.

(B) Dbf4 interacts with Ulp2 (catalytically dead ulp2-C624S, $Ulp2_{CD}$, has stronger interaction with its substrates compared to the WT protein) in the Y2H system. 8mM 3-amino-triazole (3-AT) added to reduce auto-activation of the *HIS3* reporter gene.

(C) Overlapping ChIP-on-chip profiles of Ulp2-PK and Ulp2-FLAG from cells released into the S phase in the presence of 0.2 M HU for 90 min after G1 arrest. Chromosome 12 with annotated ARS regions and centromere is shown as an example. The p values of the genome-wide overlap between the Ulp2-PK and Ulp2-FLAG clusters, as well as between the Ulp2-PK clusters in two independent experiments are indicated.

(D) BrdU IP-on-chip profiles (orange) and ChIP-on-chip profiles of Ulp2-PK (green) from cells released into the S phase in the presence of 0.2 M HU and BrdU for 90 min after G1 arrest, and from cells subsequently released into YPD plus BrdU for 30 min following HU washout. Average Ulp2-PK binding and BrdU incorporation profiles in a window of 40 kbps centered at each of the 141 active ARSs are shown (right).

(E) Ulp2 interacts with Mcm4 subunit of the MCM helicase in Co-IP studies. Whole cell extracts (WCE) were prepared from cells expressing C-terminally 9PK-tagged Ulp2 and 13MYC-tagged Mcm4. DDK-phosphorylated Mcm4 species, other MCM subunits and $Ulp2^{9PK}$ CoIP, when $Mcm4^{13MYC}$ is immunoprecipitated with anti-Myc antibody, but not with mouse IgG.

(F) ChIP-on-chip profiles of Ulp2-PK from WT cells and cells with ARSs 604-607 being mutated (*ars*Δ**)**. Entire chromosome 6 with annotated ARS regions and centromere is shown. The p value is related to the genome-wide overlap between the Ulp2-PK clusters in the two indicated strains.

(G) SUMO conjugates are enriched at the replication origins. BrdU IP-on-chip profile (orange) and ChIP-on-chip profile of Flag-SUMO (dark green) from cells released into the S phase in the presence of 0.2 M HU and BrdU, as in (D).

Figure S2

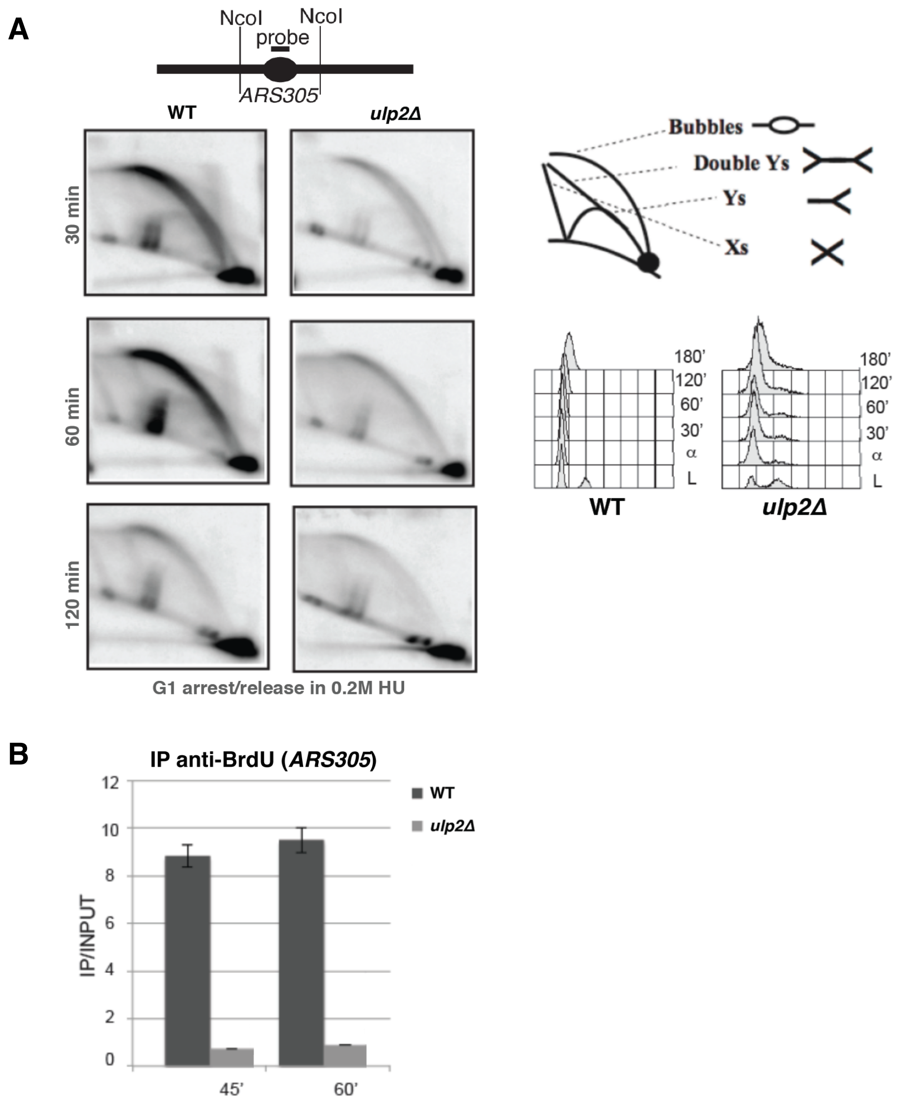

Figure S2. Accumulation of Replication Intermediates and BrdU Incorporation Are Decreased in *ulp2*Δ Cells, Related to Figure 2.

(A) The abundance of replication intermediates is decreased in cells lacking Ulp2. WT and *ulp2*Δ cells were released from G1 arrest into the S phase in the presence of 0.2 M HU for the indicated time (min), as judged from the FACS profiles (bottom right), and replication intermediates subjected to 2D gel analysis. Schematic representations of the 2D gel fragment analyzed (top) and of the type of intermediates that may be revealed by 2D gel (top right).

(B) BrdU incorporation at early replication origins is decreased in *ulp2*Δ cells compared to WT as monitored by BrdU IP-qPCR at *ARS305* in cells synchronized in G1 phase and released into the S phase in the presence of 0.2 M HU and BrdU for the indicated time (min). Error bars represent standard deviations.

Figure S3

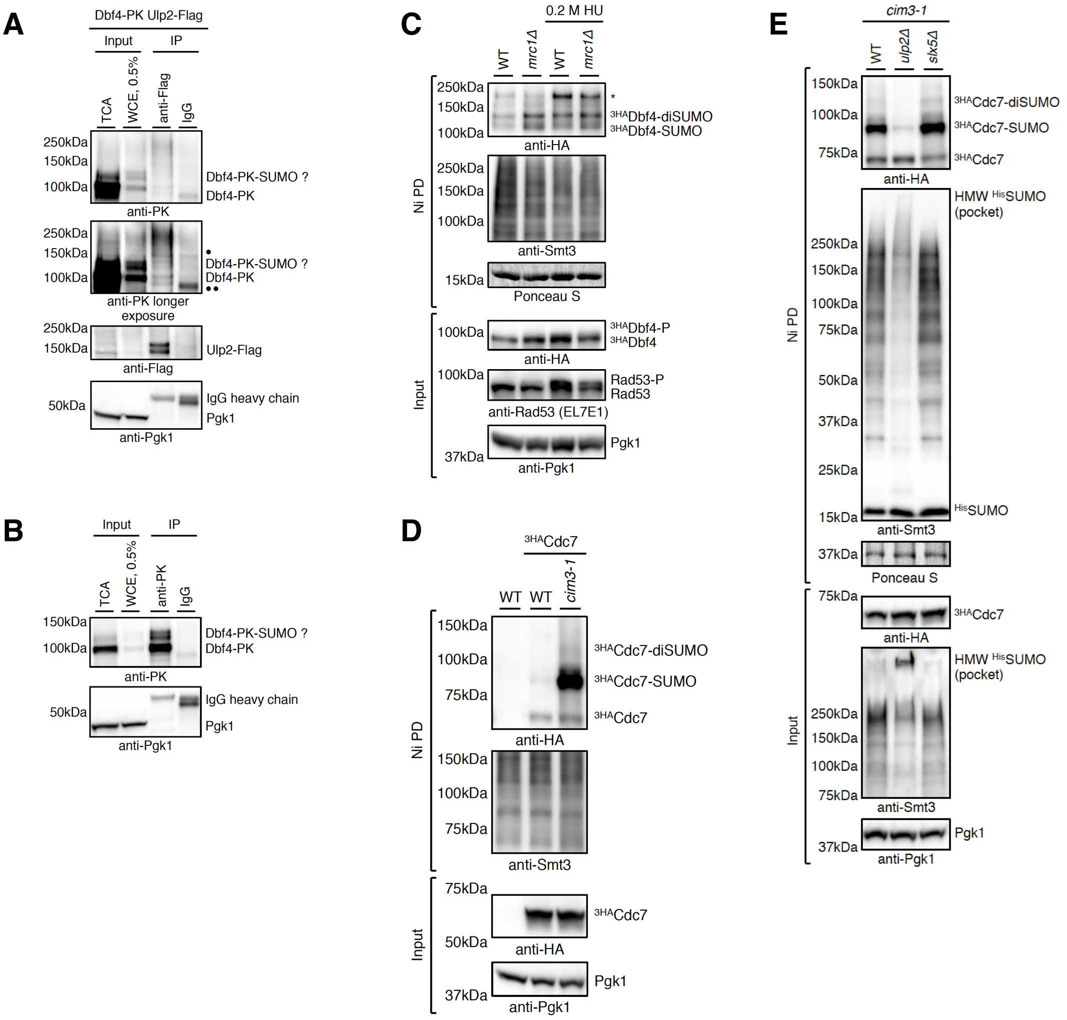

Figure S3. Chromatin-bound DDK Engaged in Replication is SUMOylated and Safeguarded by Ulp2 Against the Slx5/8 STUbL-mediated Proteasomal Degradation, Related to Figure 3.

(A) Ulp2 interacts with Dbf4 in the Co-IP studies with preference towards slower-migrating potentially SUMOylated Dbf4 species. Proteins were precipitated using trichloroacetic acid (TCA) to preserve the posttranslational modifications, or whole cell extracts (WCE) were prepared from cells expressing C-terminally 9PK-tagged Dbf4 and 3Flag-tagged Ulp2 using grinding in liquid nitrogen. Both unmodified and slower-migrating potentially SUMOylated species of Dbf4-PK Co-IP during IP with anti-Flag antibody, but not with mouse IgG. Single and double filled circles denote cross-reactivity of the anti-PK antibody.

(B) IP of slower-migrating potentially SUMOylated species of Dbf4-PK. Similar to (A), but Dbf4-PK and its modified species are immunoprecipitated specifically with anti-PK antibody and not with mouse IgG.

(C) Dbf4 SUMOylation is increased in the absence of S-phase checkpoint protein Mrc1. ^{His}SUMO Ni PD from untreated WT and *mrc1*Δ cells expressing ^{3HA}Dbf4 under the control of *ADH1* promoter, or grown to an OD_{600} of 0.7 and shifted to YPD containing 0.2 M HU for 90 min. Ni PD efficiency was assayed using anti-Smt3 antibody and staining with Ponceau S (nonspecifically-bound protein of ≈15 kDa visualized). Dbf4 phosphorylation (Dbf4-P) is lost and Rad53 phosphorylation (Rad53-P) is markedly reduced in *mrc1*Δ cells treated with HU compared to WT. Asterisk denotes cross-reactivity of the anti-HA antibody.

(D) SUMOylated Cdc7 species, but not unmodified Cdc7, strongly accumulate in the temperature sensitive *cim3-1* proteasome-defective mutant grown at permissive temperature (28°C) compared to WT cells. ^{His}SUMO Ni PD from WT and *cim3-1* cells expressing either untagged or N-terminally 3HA-tagged Cdc7 under the control of *ADH1* promoter. Unmodified forms of Cdc7 are nonspecifically pulled-down and can be detected in Ni PD.

(E) Turnover of degradation-prone monoSUMOylated Cdc7 is accelerated in the absence of Ulp2. Similar to (D), but with *cim3-1, cim3-1 ulp2*Δ*, and cim3-1 slx5*Δ cells grown at 28°C.

Figure S4

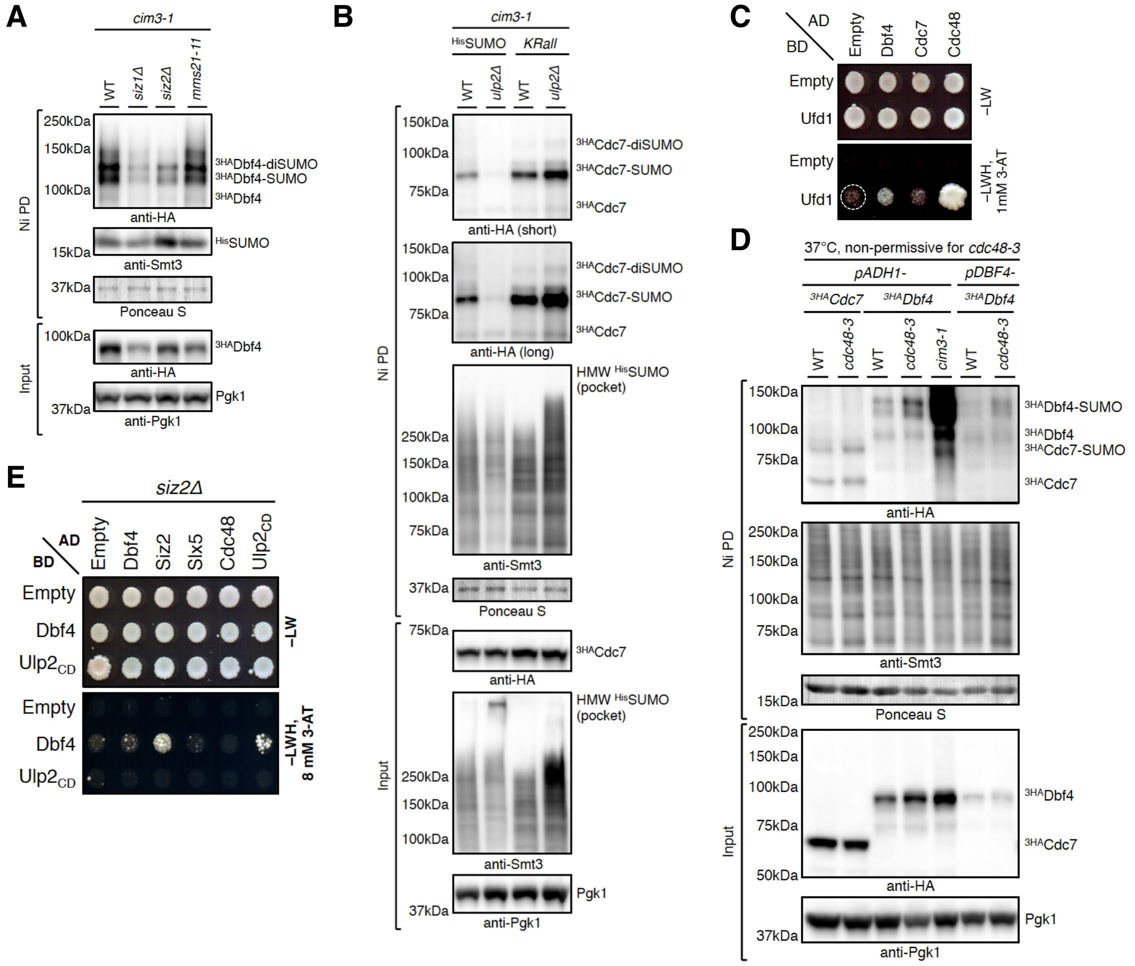

Figure S4. SUMO/Ubiquitin-dependent Segregase Cdc48 Assists in the Proteasomal Degradation of DDK Modified by SUMO Ligases Siz1 and Siz2, Related to Figure 4.

(A) SUMOylation of Dbf4 is mediated by the SUMO ligases Siz1 and Siz2. Ni PD of ^{His}SUMO conjugates from *cim3-1* mutant cells expressing ^{3HA}Dbf4 under the control of *ADH1* promoter (WT) and cells additionally lacking SUMO ligase Siz1 (*siz1*Δ), Siz2 (*siz2*Δ), or carrying *mms21-11* allele, a mutant of Mms21 that is deficient in SUMO ligase activity. Ni PD efficiency was assayed using anti-Smt3 antibody and staining with Ponceau S (nonspecifically pulled-down protein of ≈37 kDa visualized).

(B) The decreased levels of monoSUMOylated Cdc7 species in *cim3-1 ulp2*Δ mutant cells are restored if instead of ^{His}SUMO a lysine-less SUMO variant (*KRall*) that cannot form lysine-linked SUMO chains is expressed as the only source of SUMO.

(C) Dbf4 interacts with the Cdc48 segregase substrate-recruiting co-factor Ufd1 in the Y2H system. 1mM 3-amino-triazole (3-AT) added to reduce auto-activation of the *HIS3* reporter gene by BD-Ufd1 fusion alone (marked by circle).

(D) Cdc48 segregase assists in the proteasomal degradation of SUMOylated DDK. ^{His}SUMO Ni PD from WT cells and temperature sensitive *cdc48-3* mutant expressing ^{3HA}Cdc7 under the control of strong *ADH1* promoter (*pADH1*), and ^{3HA}Dbf4 either under the control of *ADH1* or endogenous promoter (*pDBF4*), grown to an OD_{600} of 0.7 at 28°C and then shifted to 37°C for 3 hours. SUMOylated DDK subunits accumulate in *cdc48-3* mutant compared to WT cells.

(E) Binding of Dbf4 to the Slx5 STUbL subunit is lost in the Y2H system, while interaction between Dbf4 and Ulp2 is strongly diminished, in the absence of the Siz2 SUMO ligase. Interaction between Ulp2 and Cdc48 is Siz2-dependent. 8mM 3-AT used to reduce background growth.

Figure S5

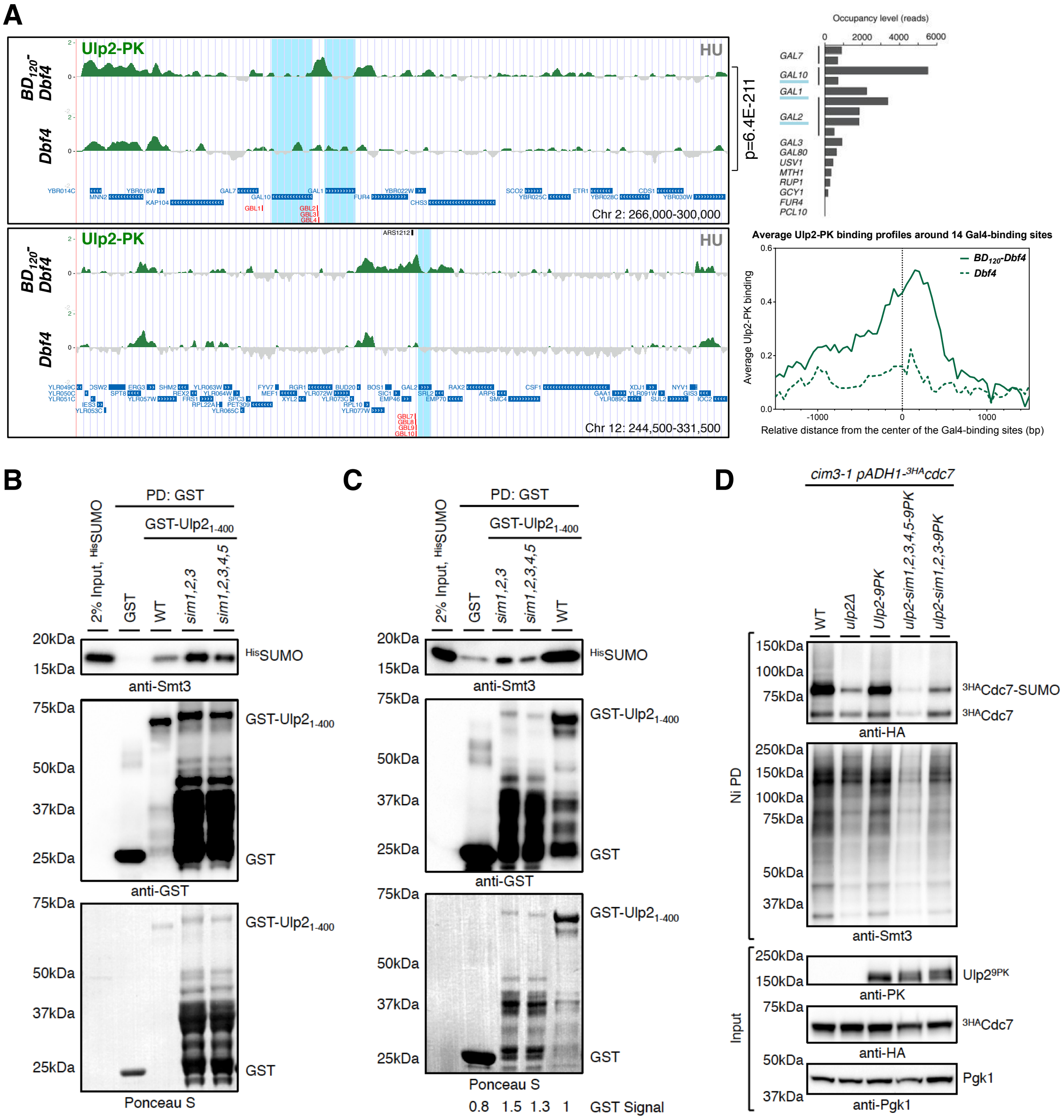

Figure S5. Ulp2 Is Recruited to Chromatin-bound SUMOylated DDK Artificially Targeted to Gal4-binding Sites and Uses Its N-terminal SIMs for Interaction, Related to Figure 5.

(A) N-terminal fusion of the Gal4 transcriptional activator DNA-binding domain to Dbf4 (BD_{120}-Dbf4) recruits Ulp2 to the Gal4-binding locations (GBL) at the genes of the yeast galactose regulon. ChIP-on-chip profiles of Ulp2-PK from cells expressing either N-terminally 3HA-tagged Dbf4 or BD_{120}-Dbf4 fusion under the control of *ADH1* promoter released into the S phase in the presence of 0.2 M HU for 90 min. Fragments of chromosomes 2 and 12 with the annotated GBLs in red and the

GAL genes with the highest Gal4 occupancy highlighted in blue are shown (left). The p value is related to the genome-wide overlap between the Ulp2-PK clusters in the two indicated strains. Average Ulp2-PK binding profiles in a window of 3 kbps centered at 14 GBLs (right).

(B-C) Recombinant GST-Ulp2$_{1-400}$ fusions with either first three (*sim1,2,3*) or all five (*sim1,2,3,4,5*) potential N-terminal SIMs mutated form multiple degradation products compared to WT GST-Ulp2$_{1-400}$ when expressed in *E. coli* that retain ability to pull-down free ^{His}SUMO, albeit with strongly reduced affinity (close to background in case of *sim1,2,3,4,5*).

(D) Both *ulp2-sim* mutants fail to protect monoSUMOylated Cdc7 against SUMO-chain/STUbL-mediated proteasomal degradation similar to *ulp2*Δ cells. ^{His}SUMO Ni PD from *cim3-1*, *cim3-1 ulp2*Δ, and *cim3-1* cells carrying either Ulp2-9PK or its SIM-mutant variants that express ^{3HA}Cdc7 under the control of *ADH1* promoter. monoSUMOylated Cdc7 species strongly accumulate in *cim3-1* and *cim3-1 Ulp2-9PK*, but not in *cim3-1 ulp2* double mutants. Unmodified Cdc7 is nonspecifically pulled-down and is detected below SUMO-modified species in Ni PD.

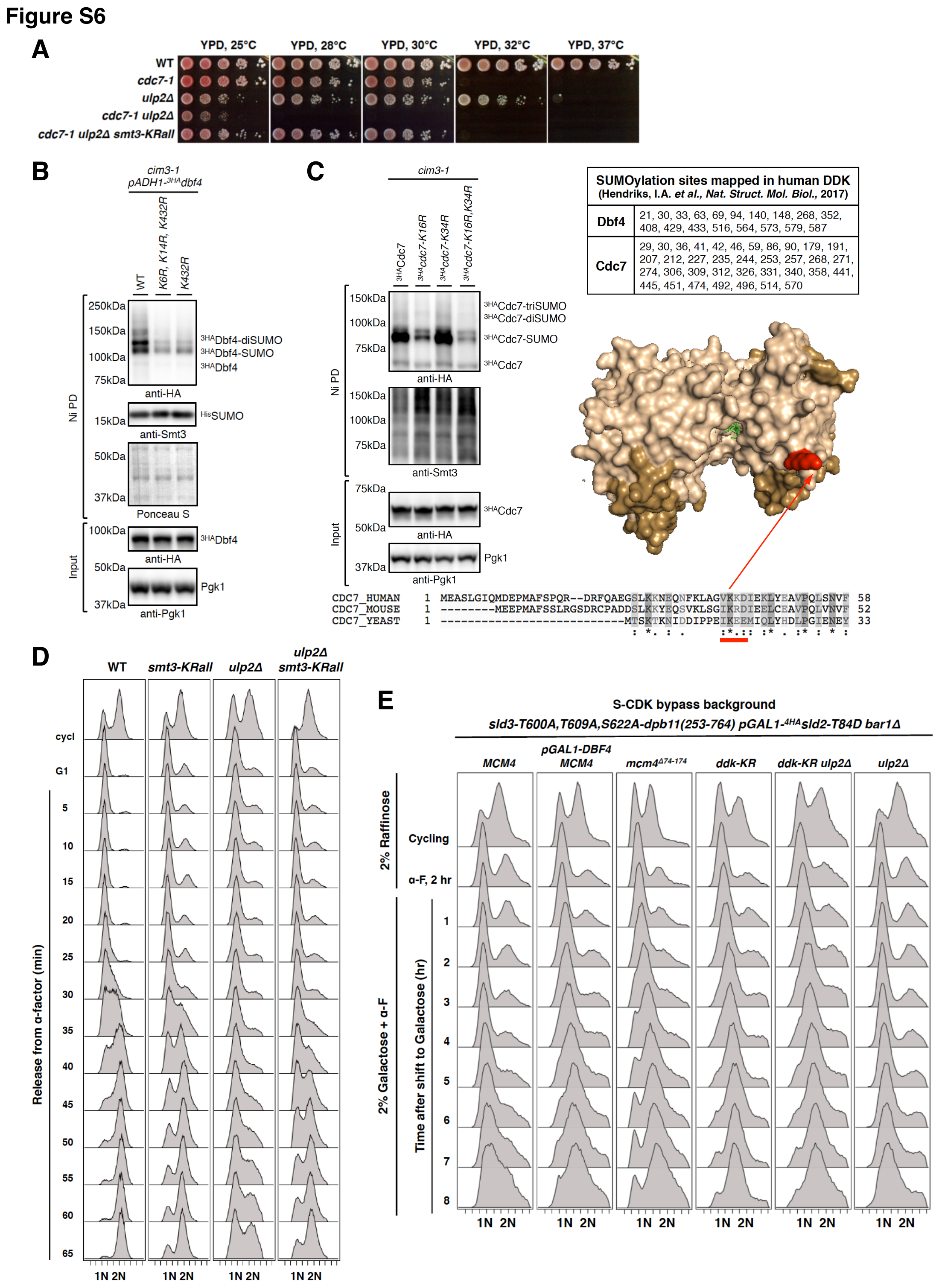

Figure S6. SUMOylation-defective *ddk-KR* Mutant No Longer Requires Ulp2 For Protection Against STUbL-mediated Proteasomal Degradation and Efficiently Promotes Replication Onset, Related to Figure 6.

(A) Temperature sensitive *cdc7-1* mutant exhibits synthetic lethal genetic interaction with *ulp2*Δ at permissive temperatures for *cdc7-1* cells that can be suppressed by *smt3-KRall* mutation. Spotting of 1:7 serial dilutions on YPD plates at indicated temperatures.

(B-C) Mapping of major SUMO acceptor sites in DDK and generation of a SUMOylation-defective *ddk-KR* mutant. Major SUMO acceptor lysines (K) were identified and replaced by arginines (R) to abolish the modification. ^{His}SUMO Ni PD from *cim3-1* proteasome-defective mutant cells expressing either N-terminally 3HA-tagged Dbf4 (B) or Cdc7 (C) and their various KR variants. Both DDK subunits are SUMOylated at multiple lysines, each contributing to a different extent to the overall DDK SUMOylation, as in the case of human DDK SUMOylation (C, above right). Cdc7 is largely SUMOylated at conserved K16. Structure of the human Cdc7-Dbf4 heterodimer (Hs_Cdc7, wheat; Hs_Dbf4, sand; C, below right) with ADP (green) modeled at active site. Validated SUMO acceptor lysine 16 (K16) in budding yeast Cdc7 lies within a SUMO consensus motif **ψ-K-x-E/D** (ψ, a hydrophobic amino acid; x, any amino acid), which is conserved in mouse and human (underlined red in the alignment of the Cdc7 N-termini from budding yeast, mouse and human), and corresponds to K41 in human Cdc7 (highlighted in red in the structure).

(D) S phase progression defect in *ulp2*Δ cells is suppressed by *smt3-KRall*. Exponentially growing WT, *smt3-KRall*, *ulp2*Δ, and *ulp2*Δ *smt3-KRall* cells (cycl) arrested in G1 phase by α-factor, released into YPD at 25°C, and samples taken every 5 minutes for FACS.

(E) Replication initiation defect in *ulp2*Δ S-CDK bypass background cells can be suppressed by the SUMOylation-defective *ddk-KR* mutant. Exponentially growing (Cycling) S-CDK bypass background cells (*MCM4*), *MCM4* cells harboring extra copy of *DBF4* under the control of galactose-inducible promoter (*pGAL1-DBF4*), *mcm4*$^{\Delta 74\text{-}174}$, *ddk-KR, ulp2*Δ, and *ulp2*Δ *ddk-KR* cells cultured in YP raffinose (2%) were arrested in G1 phase by α-factor (α-F), rapidly switched to YP galactose (2%) containing α-F at 28°C, and samples were taken every hour for FACS analysis.

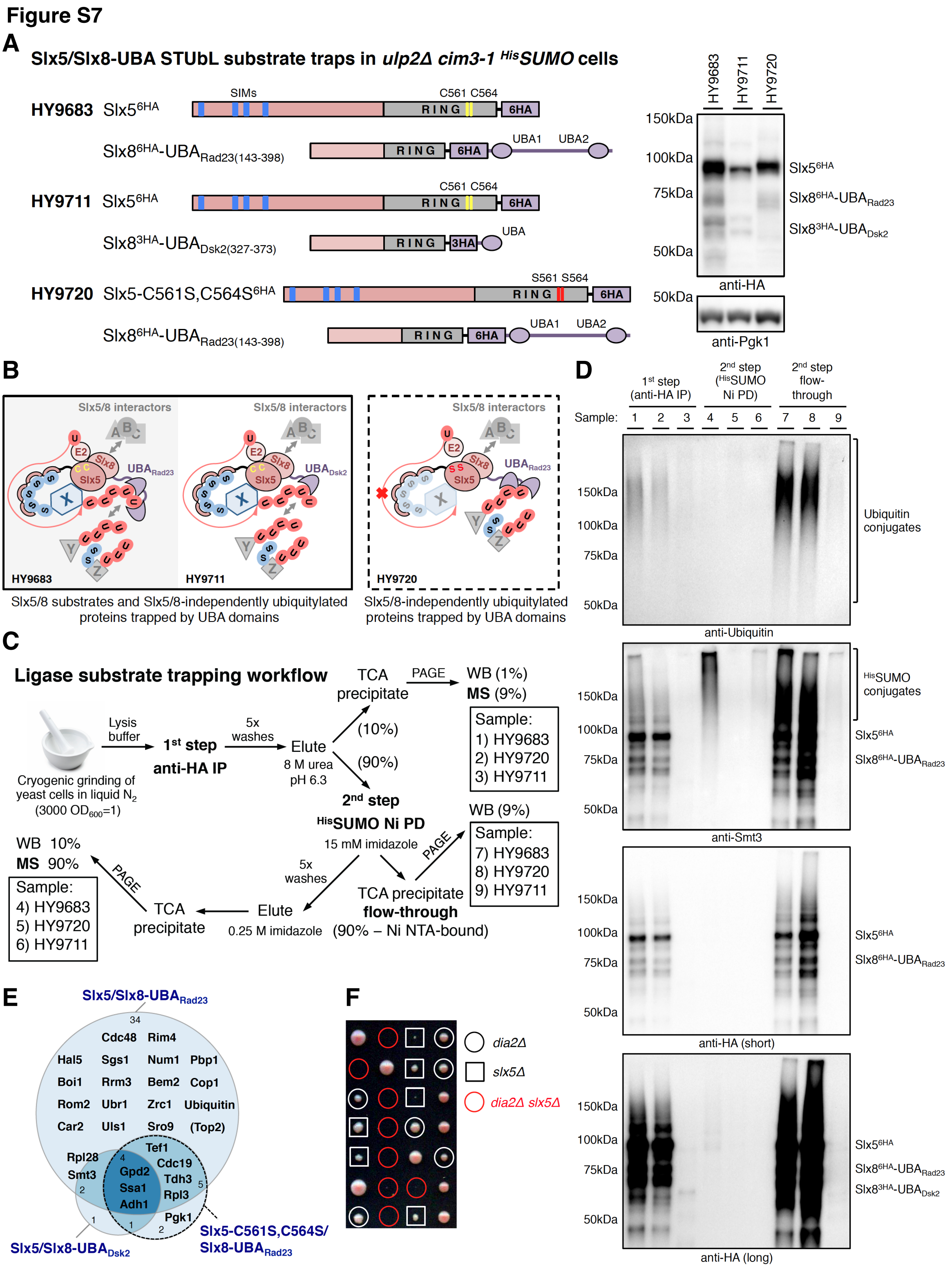

Figure S7. Slx5/8 STUbL Substrate Trapping in *cim3-1 ulp2*Δ *HisSUMO* Background, Related to Figure 7.

(A) Schematic representation of the Slx5/Slx8-UBA substrate traps generated (strains HY9683, HY9711, HY9720) in *cim3-1 ulp2*Δ ^{His}*SUMO* background (left). UBA domains with different affinity towards ubiquitin chains derived either from Rad23 (aa 143-398; UBA1 and UBA2) or Dsk2 (aa 327-373) ubiquitin receptors were fused C-terminally to Slx8 via 6HA- or 3HA-containing linker sequences, respectively. Additionally, Slx5 was C-terminally tagged with 6HA in all strains with the only difference that in HY9720 point mutations (*slx5-C561S, C564S*) were introduced in the RING domain of Slx5 inactivating the Slx5/8 STUbL. Fusion of the Dsk2-derived UBA domain via 3HA to Slx8 destabilized the Slx5/8 STUbL (right).

(B) Schematic representation of the proteins predicted to bind to the generated substrate traps during the anti-HA IP. Catalytically active Slx5/Slx8-UBA traps may co-IP Slx5/8 interactors (labeled A, B, C), Slx5/8-independently ubiquitylated proteins (labeled Y, Z) and true Slx5/8 substrates (labeled X) in most cases marked with SUMO chains, whereas catalytically inactive slx5-C561S, C564S/Slx8-UBA fusion can only trap interactors and Slx5/8-independently ubiquitylated proteins, thus serving as a nonspecific background binding control.

(C) Schematic representation of the Slx5/8 ligase substrate trapping workflow and samples (1-9) taken for western blot (WB) procedure control (see panel D) or mass spectrometry (MS) analysis. Percentage in parentheses represents amount of immunoprecipitated material following 1st step anti-HA IP taken for subsequent steps: WB control, MS, 2nd step Ni-NTA pull-down (Ni PD) of ^{His}SUMO conjugates.

(D) Western blot control of the Slx5/8 ligase substrate trapping procedure. Both catalytically active and inactive stable Slx5/Slx8-UBA$_{Rad23}$ traps efficiently co-IP ubiquitin conjugates (samples 1, 2, 7, 8; anti-Ubiquitin), whereas SUMO conjugates – expected substrates of STUbL – are enriched after 2nd step ^{His}SUMO Ni PD only in strains expressing catalytically active Slx5 (samples 4 and 6; anti-Smt3), even despite of the low stability of Slx5/Slx8-UBA$_{Dsk2}$ trap (samples 3 and 9; anti-HA), but not when Slx5/8 STUbL is inactive (sample 5; anti-Smt3).

(E) Euler diagram representing proteins detected by mass spectrometry following 2nd step ^{His}SUMO Ni PD of the Slx5/8 STUbL substrate trapping procedure (samples 4, 5, 6 in panels C and D). Proteins (see also Table S1) were identified using Scaffold software with stringent criteria; Top2 (in parentheses) was detected with one unique peptide.

(F) Mutations of the F-box protein Dia2 that drives MCM helicase ubiquitylation during DNA replication termination (*dia2*Δ) and of the Slx5/8 STUbL (*slx5*Δ) are synthetically lethal. Tetrad dissection of the *DIA2*/*dia2*Δ *SLX5*/*slx5*Δ diploid yeast.
