# Supplementary material for: SUMO-Chain-Regulated Proteasomal Degradation Timing Exemplified in DNA Replication Initiation
Source: Mol Cell. 2019 Nov 21;76(4):632–645.e6. doi: 10.1016/j.molcel.2019.08.003 (PMC6891891; doi:10.1016/j.molcel.2019.08.003)
Supplement: Document S2. Article plus Supplemental Information [file mmc4.pdf]

Article

Molecular Cell

SUMO-Chain-Regulated Proteasomal Degradation Timing Exemplified in DNA Replication Initiation

Graphical Abstract

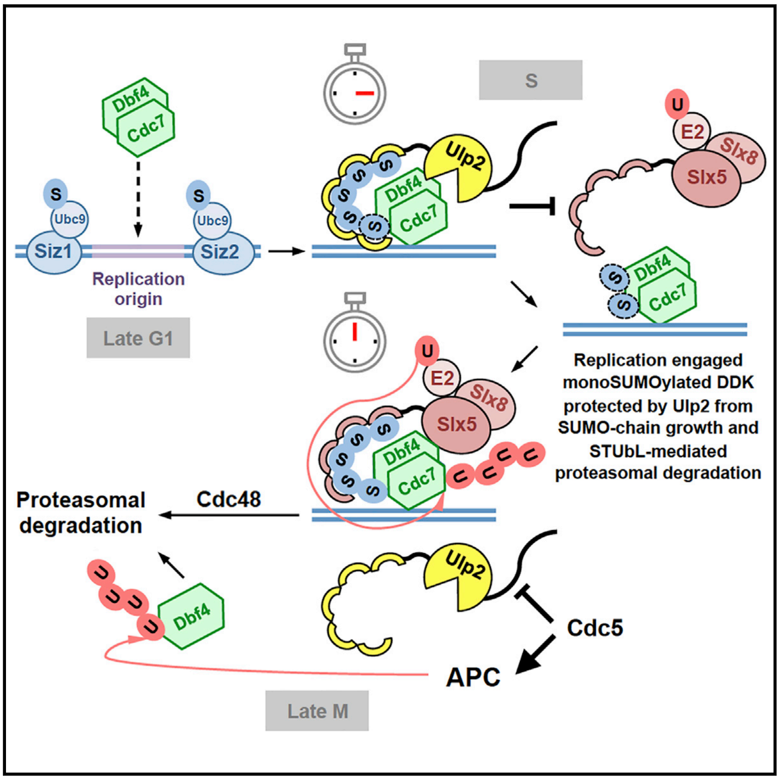

Authors

Ivan Psakhye, Federica Castellucci, Dana Branzei

Correspondence

dana.branzei@ifom.eu

In Brief

Psakhye et al. uncover that SUMO chains specifically counteracted by the Ulp2 SUMO protease function like a countdown timer when they are assembled on substrates of the Slx5/Slx8 SUMO-targeted ubiquitin ligase (STUbL), which channels them for proteasomal degradation, as demonstrated here for SUMOylated DDK engaged in DNA replication initiation.

Highlights

- Chromatin-bound DDK engaged in replication is SUMOylated at multiple sites
- SUMO chains target DDK for STUbL-mediated Cdc48-assisted proteasomal degradation
- SUMO protease Ulp2 protects DDK from degradation, allowing early steps of replication
- Ulp2 curbs SUMO chain assembly, thus timing protein turnover in replication and beyond

Psakhye et al., 2019, Molecular Cell *76*, 632–645
November 21, 2019 © 2019 The Author(s). Published by Elsevier Inc.
https://doi.org/10.1016/j.molcel.2019.08.003

CellPress

Molecular Cell
Article

SUMO-Chain-Regulated Proteasomal Degradation Timing Exemplified in DNA Replication Initiation

Ivan Psakhye,[1] Federica Castellucci,[1] and Dana Branzei[1,2,3,*]
[1]IFOM, FIRC Institute of Molecular Oncology, Via Adamello 16, 20139 Milan, Italy
[2]Istituto di Genetica Molecolare, Consiglio Nazionale delle Ricerche (IGM-CNR), Via Abbiategrasso 207, 27100 Pavia, Italy
[3]Lead Contact
*Correspondence: dana.branzei@ifom.eu
https://doi.org/10.1016/j.molcel.2019.08.003

SUMMARY

Similar to ubiquitin, SUMO forms chains, but the identity of SUMO-chain-modified factors and the purpose of this modification remain largely unknown. Here, we identify the budding yeast SUMO protease Ulp2, able to disassemble SUMO chains, as a DDK interactor enriched at replication origins that promotes DNA replication initiation. Replication-engaged DDK is SUMOylated on chromatin, becoming a degradation-prone substrate when Ulp2 no longer protects it against SUMO chain assembly. Specifically, SUMO chains channel DDK for SUMO-targeted ubiquitin ligase Slx5/Slx8-mediated and Cdc48 segregase-assisted proteasomal degradation. Importantly, the SUMOylation-defective *ddk-KR* mutant rescues inefficient replication onset and MCM activation in cells lacking Ulp2, suggesting that SUMO chains time DDK degradation. Using two unbiased proteomic approaches, we further identify subunits of the MCM helicase and other factors as SUMO-chain-modified degradation-prone substrates of Ulp2 and Slx5/Slx8. We thus propose SUMO-chain/Ulp2-protease-regulated proteasomal degradation as a mechanism that times the availability of functionally engaged SUMO-modified protein pools during replication and beyond.

INTRODUCTION

Reversible covalent modification of proteins with the small ubiquitin-like modifier (SUMO) is critical for the viability of eukaryotic cells by affecting protein interactions, sub-cellular localization, and activity (Flotho and Melchior, 2013). SUMO is conjugated to acceptor lysines on the surface of its substrates in the form of monomers, leading to monoSUMOylation or multiSUMOylation when several target lysines are modified. SUMOylation frequently targets entire protein groups actively engaged in common functions (Jentsch and Psakhye, 2013; Psakhye and Jentsch, 2012). Moreover, various proteins contain SUMO-interacting motifs (SIMs) with which they can engage in non-covalent interactions with SUMO (Song et al., 2004), stabilizing protein assemblies. Similar to ubiquitin, SUMO can form polymeric chains via different linkages, leading to substrate polySUMOylation; however, functional insights into the role of SUMO chains are limited (Flotho and Melchior, 2013; Srikumar et al., 2013; Vertegaal, 2010). Both mono- and/or multiSUMOylation and polySUMO chains can be further recognized by ubiquitin E3 ligases containing single or multiple SIMs. These ubiquitin ligases, known as SUMO-targeted ubiquitin ligases (STUbLs), can mediate proteolytic or non-proteolytic ubiquitylation of the SUMO-modified substrate (Sriramachandran and Dohmen, 2014). The outcome depends, in manners that remain largely to be deciphered, on both the substrate and the STUbL involved. Moreover, ubiquitin and SUMO attached to the substrate can cooperate to recruit downstream factors, such as the Cdc48/p97 ATPase (Bergink et al., 2013; Dantuma and Hoppe, 2012; Maric et al., 2014), that decide the fate of the modified substrate.

SUMO chains are thought to have signaling functions in the cell, as for synaptonemal complex assembly in budding yeast meiosis (Cheng et al., 2006) and replication arrest response in fission yeast (Skilton et al., 2009). SUMO chains have also been suggested to play roles in chromatin regulation, including maintenance of higher-order chromatin structure and transcriptional repression of environmental stress response genes in budding yeast (Srikumar et al., 2013). This is likely based on the ability of SUMO chains to attract various SIM-containing factors or STUbLs or become disassembled by specific SUMO proteases; namely, SENP6/SENP7 in mammalian cells and Ulp2 in budding yeast (Bylebyl et al., 2003; Eckhoff and Dohmen, 2015; Hickey et al., 2012). However, the role of SUMO chains in signaling remains largely enigmatic, and much remains to be understood about the identity of SUMO substrates that require SUMO chains for proteasomal degradation or other functions.

Here we attempted to address the molecular function of SUMO chains by focusing on the budding yeast SUMO protease Ulp2, which provides the main SUMO-chain-depolymerizing activity in this organism. Using a yeast two-hybrid (Y2H) screen, we identified Dbf4, the DNA-binding subunit of Dbf4-dependent kinase (DDK), which mediates DNA replication initiation by phosphorylating the replicative helicase MCM (minichromosome

 © 2019 The Author(s). Published by Elsevier Inc.
This is an open access article under the CC BY license (http://creativecommons.org/licenses/by/4.0/).

maintenance protein complex) (Bell and Labib, 2016), as an interactor of Ulp2. We uncovered that Ulp2 is enriched at replication origins and that its loss leads to inefficient replication onset, which can be rescued by concomitant inactivation of the Slx5/8 STUbL. We found that a critical substrate for Ulp2 and Slx5/8 in this process is DDK itself. Both subunits of DDK, Dbf4 and Cdc7, which are actively engaged in replication, become SUMOylated on chromatin. Ulp2 protects mono- and/or multiSUMOylated DDK against Slx5/8-mediated proteasomal degradation by binding to growing SUMO chains via multiple SIMs located in its N terminus and trimming them from the distal ends. The proteasome-mediated turnover of SUMOylated DDK is assisted by the Cdc48 segregase and strictly depends on SUMO chains that are counteracted by Ulp2. Importantly, the SUMOylation-defective *ddk-KR* mutant, in which major SUMO acceptor lysines are replaced with arginines on both Dbf4 and Cdc7, suppresses the replication onset defects and rescues reduced Mcm4 phosphorylation and slower S phase progression of the *ulp2Δ* mutant. Altogether, the results indicate that Ulp2 allows critical levels of mono- and/or multiSUMOylated DDK to be locally concentrated at origins of replication, making efficient onset of replication possible. Using two unbiased Slx5/8 ubiquitin ligase substrate trapping and stable isotope labeling with amino acids in cell culture (SILAC)-based proteomic screens, we further identify subunits of the MCM helicase and other factors as potential SUMO-chain-modified degradation-prone substrates of Ulp2 and Slx5/Slx8. We propose that SUMO-chain- and Ulp2-protease-regulated proteasomal degradation acts as a fate maker of protein availability at replication origins and, possibly, in other replication and cellular contexts.

RESULTS

The SUMO Protease Ulp2 Binds to Dbf4 and Is Enriched at Replication Origins

In a Y2H screen using the Ulp2 SUMO protease as bait, we identified the Dbf4 subunit of DDK and the Polo-like kinase Cdc5 as Ulp2 interactors (Figure S1A). Because Ulp2 has been reported previously to interact with and be negatively regulated by Cdc5 in mitosis (Baldwin et al., 2009), here we pursued the interaction between Ulp2 and Dbf4, which we further confirmed by Y2H analysis in both orientations (Figure S1B). We used catalytically dead transcription activation domain (AD)-fusion and DNA-binding domain (BD)-fusion of Ulp2 (Ulp2-C624S; $Ulp2_{CD}$), which we envisaged to interact stronger with potential substrates based on previous work on $Ulp1_{CD}$, which behaved like a SUMO substrate trap (Elmore et al., 2011). To examine whether Ulp2 binds to chromatin and map its clusters, we used genome-wide chromatin immunoprecipitation (ChIP) studies (ChIP-on-chip) in G1-arrested and hydroxyurea (HU)-treated wild-type (WT) cells expressing endogenously tagged Ulp2-PK. Multiple chromatin-bound clusters of Ulp2 were detected across the genome under both conditions. Importantly, we found that Ulp2 binds to autonomously replicating sequence (ARS) regions marking origins of replication in both G1 and S phase (Figure 1A) in a manner that is statistically significant ($p = 5.1E-19$ and $p = 5.6E-67$ for G1 and S, respectively). The peaks of Ulp2 proximal to early ARS regions were wider in HU-treated cells compared with G1 (Figure 1A), indicating spreading of Ulp2 around active replication origins. A nearly identical profile of Ulp2 binding to chromatin was observed with the endogenously tagged Ulp2-FLAG strain (Figure S1C).

We further found genome-wide statistically significant overlap between Ulp2 and bromodeoxyuridine (BrdU) clusters that mark ongoing replication, with both Ulp2 and BrdU peaks centered on origins of replication in cells released from G1 arrest into HU- and BrdU-containing media (Figure 1B). Moreover, when cells were released from HU arrest into medium containing BrdU, Ulp2 peaks became broader compared with those in HU (Figure S1D), indicating that Ulp2 may bind replication fork components. Supportive of this, we found, by co-immunoprecipitation (coIP), that Ulp2 interacts with the Mcm4 subunit of the MCM helicase (Figure S1E).

To address whether Ulp2 binding to replicating regions depends on ARS elements, we used a strain in which three early ARS regions in chromosome 6 were abolished (*arsΔ* strain) (Dershowitz and Newlon, 1993). Ulp2 binding specifically to the mutated ARS regions was lost (Figures 1C and S1F). Regarding the potential mechanism implicated in Ulp2 recruitment, we found that yeast SUMO N-terminally tagged with FLAG (FLAG-SUMO) was also enriched at replicating regions incorporating BrdU (Figure S1G). Altogether, the results suggest that Ulp2 is recruited to replication origins, likely by its ability to recognize SUMOylated replisome factors.

Ulp2 Allows Efficient Replication Onset by Counteracting Slx5/8 STUbL

To determine the consequences of Ulp2 recruitment to replication origins, we examined, by 2D DNA gel electrophoresis, the profile of replication intermediates formed at an early efficient origin of replication, ARS305, when G1-arrested cells were synchronously released in HU-containing medium. The density of replication intermediates, bubbles and Y-arcs, was reduced in *ulp2Δ* cells (Figure S2A). Moreover, we observed reduced BrdU incorporation efficiency in *ulp2Δ* genome-wide (Figures 2A and 2B) and at ARS305 measured by BrdU immunoprecipitation (IP)-qPCR (Figure S2B). Thus, *ulp2Δ* cells have reduced efficiency in origin firing and/or early steps of DNA replication elongation.

Ulp2 has a preference for substrates modified with SUMO chains, which Ulp2 cleaves sequentially from the chain end (Eckhoff and Dohmen, 2015). Importantly, SUMOylation of certain substrates can be recognized by STUbLs that can ubiquitylate the substrate, targeting it for proteasomal degradation (Sriramachandran and Dohmen, 2014). Because some phenotypes of *ulp2Δ* cells are alleviated by concomitant deletion of heterodimeric Slx5/8 STUbL (Mullen et al., 2011), we examined whether the replication defects associated with Ulp2 loss are compensated upon inactivation of Slx5/8 by comparing the genome-wide BrdU incorporation profiles in *ulp2Δ* and *ulp2Δ slx5Δ* (Figure 2A). Averaging the BrdU incorporation profiles around 141 active replication origins revealed that the *ulp2Δ* mutation particularly affects the spreading of BrdU around ARS regions and that *slx5Δ* suppressed this phenotype (Figure 2B). These data suggest that the replication defect of *ulp2Δ* may be caused by the Slx5/8 STUbL-mediated proteasomal degradation of certain SUMOylated factor(s) required for early steps of DNA replication.

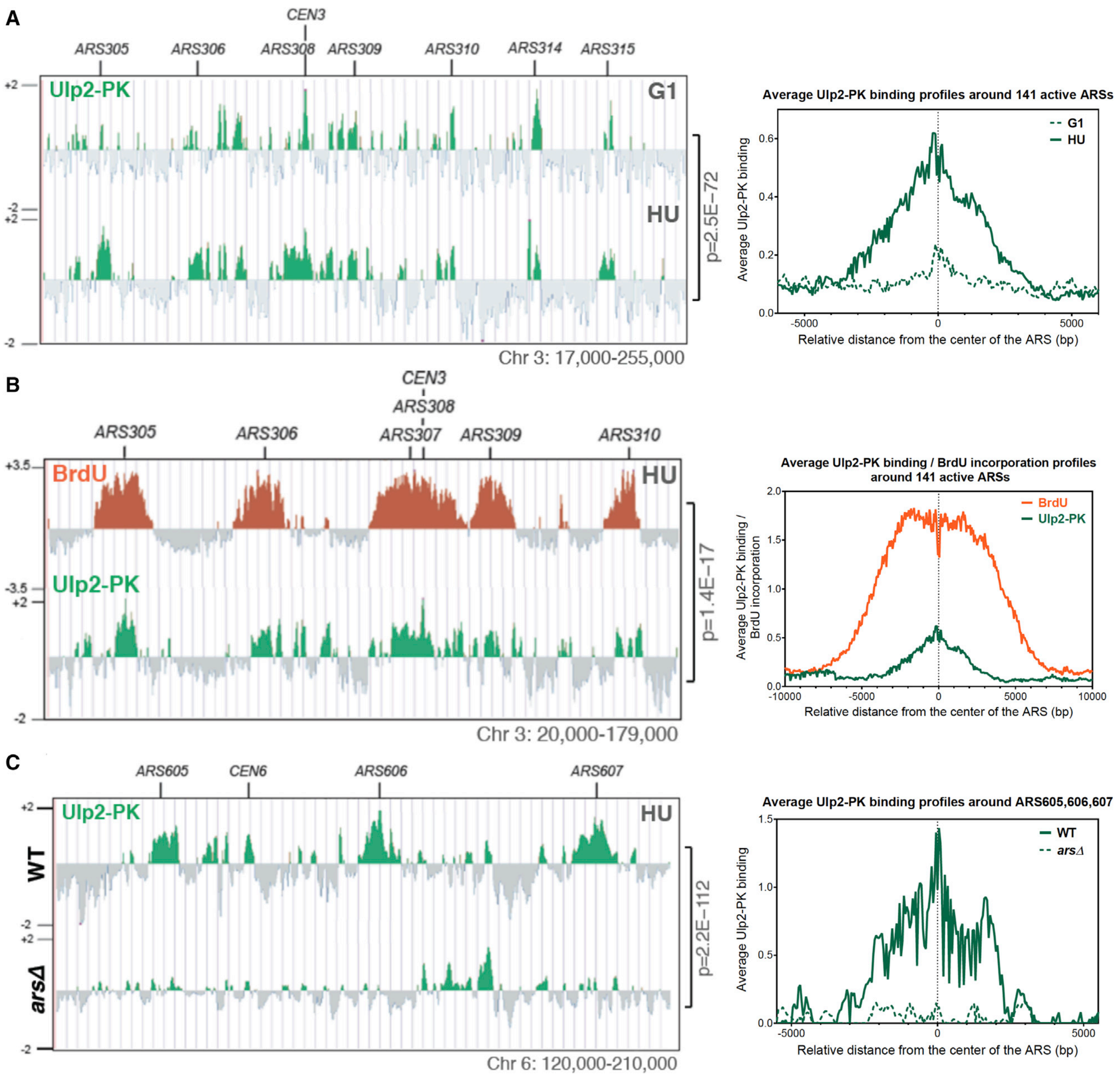

Figure 1. The SUMO Protease Ulp2 Is Enriched at Replication Origins

(A) ChIP-on-chip profiles of Ulp2-PK from G1-arrested cells following their release in the presence of 0.2 M HU for 90 min. A fragment of chromosome 3 is shown as an example (left). The p value is related to the genome-wide overlap between Ulp2-PK clusters under the two conditions. Average Ulp2-PK binding profiles in a window of 12 kbp centered at each of the 141 active ARSs are shown (right).

(B) Overlapping BrdU IP-on-chip profile (orange) and ChIP-on-chip profile of Ulp2-PK (green) from cells released in the presence of 0.2 M HU and BrdU for 90 min after G1 arrest.

(C) ChIP-on-chip profiles of Ulp2-PK from wild-type (WT) cells and cells with the indicated ARSs deleted (*ars*Δ).

See also Figure S1.

DDK Engaged in Replication Is SUMOylated and Protected by Ulp2 against Slx5/8 STUbL

We sought to validate the Ulp2 Y2H interaction with Dbf4 (Figure S1B) using coIP. Therefore, we C-terminally tagged endogenous Dbf4 and Ulp2 with PK and FLAG tags, respectively. Initial IP with the anti-FLAG antibody revealed that Ulp2-FLAG interacts with Dbf4-PK and has a preference for upshifted, potentially SUMO-modified forms of Dbf4-PK (Figure S3A). These slower-migrating species of Dbf4-PK were specifically immunoprecipitated with the anti-PK antibody but not mouse immunoglobulin G (IgG) (Figure S3B). To directly address whether Dbf4 is SUMOylated, we performed pull-down of all SUMO conjugates

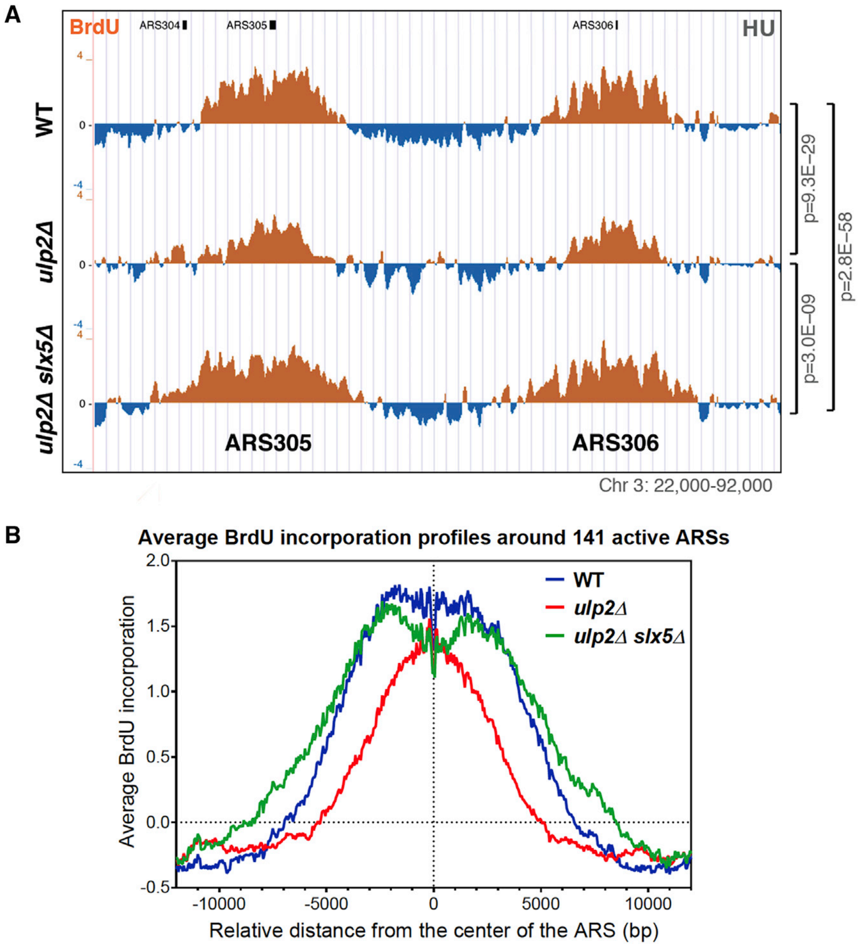

Figure 2. Ulp2 Supports Efficient Replication Onset by Counteracting the SUMO-Targeted Ubiquitin Ligase Slx5/8

(A) The decrease in BrdU incorporation in *ulp2*Δ is suppressed by deleting *SLX5*. Shown are BrdU IP-on-chip profiles from WT, *ulp2*Δ, and *ulp2*Δ *slx5*Δ cells released from G1 arrest in the presence of 0.2 M HU for 90 min.

(B) Average BrdU incorporation profiles in cells from (A) in a window of 24 kbp centered at each of the 141 active ARSs.

See also Figure S2.

present in the cell under fully denaturing conditions and probed for Dbf4. The employed strains had the endogenous yeast SUMO (Smt3) N-terminally tagged with a 7His tag (^{His}SUMO) so that SUMOylated species were enriched by nickel-nitrilotriacetic acid (Ni-NTA) pull-down (Ni PD) (Psakhye and Jentsch, 2012, 2016). We engineered cells that express N-terminally 3x hemagglutinin epitope tag (3HA)-tagged Dbf4 either under the control of the endogenous *DBF4* promoter (*pDBF4*) or a strong constitutive *ADH1* promoter (*pADH1*). Slower-migrating forms of ^{3HA}Dbf4 were specifically detected in cells expressing ^{His}SUMO and were more abundant when *DBF4* was overexpressed (Figure 3A). Thus, Dbf4 is SUMOylated.

We next inquired about the functional context in which Dbf4 undergoes SUMOylation. DDK is required for activation of all origins, but late origin firing is inhibited by the Mrc1-Rad53 checkpoint (Chen et al., 2013; Poli et al., 2012; Rossi et al., 2015a). Ni PD in *sml1*Δ and *rad53*Δ *sml1*Δ strains (*sml1*Δ allows viability of *rad53*Δ cells) revealed that Rad53 loss caused an increase in Dbf4 SUMOylation irrespective of HU treatment (Figure 3B). Thus, rather than being induced at stalled and collapsed forks, SUMOylation engages the chromatin-bound DDK pool acting at replication origins. Similar results were observed in the *mrc1*Δ mutant (Figure S3C).

To probe the importance of Dbf4 being associated with chromatin for its SUMOylation, we induced artificial DNA targeting of Dbf4 by fusing two forms of the Gal4 transcription factor DNA-binding domain to the N terminus of Dbf4 expressed from the *ADH1* promoter. One is a dimerization- and DNA binding-proficient variant spanning the 1–120 amino acids of Gal4 (BD_{120}), and the other is a dimerization- and DNA binding-defective variant spanning the 1–60 amino acids (BD_{60}) (Marmorstein et al., 1992). Ni PD assays revealed that specifically the BD_{120} fusion induces Dbf4 SUMOylation (Figure 3C). When Dbf4 was targeted to chromatin, we also detected increased SUMOylation of endogenous Cdc7 tagged C-terminally with a 9PK tag (Figure 3D). Thus, both Dbf4 and Cdc7 are SUMOylated when performing their function on chromatin.

To address whether SUMOylated DDK becomes a degradation-prone substrate of Slx5/8 STUbL that has to be protected by Ulp2 to fulfill its functions, we performed Ni PD using a temperature-sensitive *cim3-1* proteasome-defective mutant under the permissive temperature of 28°C, at which proteasomal substrates are partially stabilized. The experiments revealed strong accumulation of SUMOylated ^{3HA}Dbf4 and ^{3HA}Cdc7 species in the *cim3-1* background compared with the WT but not of their unmodified forms (Figures 3E and S3D), suggesting that specifically the SUMOylated DDK pool becomes susceptible to proteasomal degradation. Moreover, SUMOylated Dbf4 and Cdc7 species accumulating in *cim3-1* required Ulp2 for stabilization (Figures 3E and S3E). In the absence of Ulp2, high-molecular-weight (HMW) SUMO conjugates accumulate (Figures 3E and S3E; see input with HMW ^{His}SUMO conjugates; Bylebyl et al., 2003; Uzunova et al., 2007). These HMW SUMO conjugates were not detected in the corresponding Ni PD, suggesting that they are lost during the pull-down procedure. This likely explains both the apparent decrease in Ni PD efficiency of ^{His}SUMO conjugates from *ulp2*Δ cells and the inability to detect SUMO-chain-modified Dbf4 and Cdc7 in *cim3-1 ulp2*Δ cells. Importantly, the decreased levels of degradation-prone SUMOylated Dbf4 in *cim3-1 ulp2*Δ cells were restored in *cim3-1 ulp2*Δ

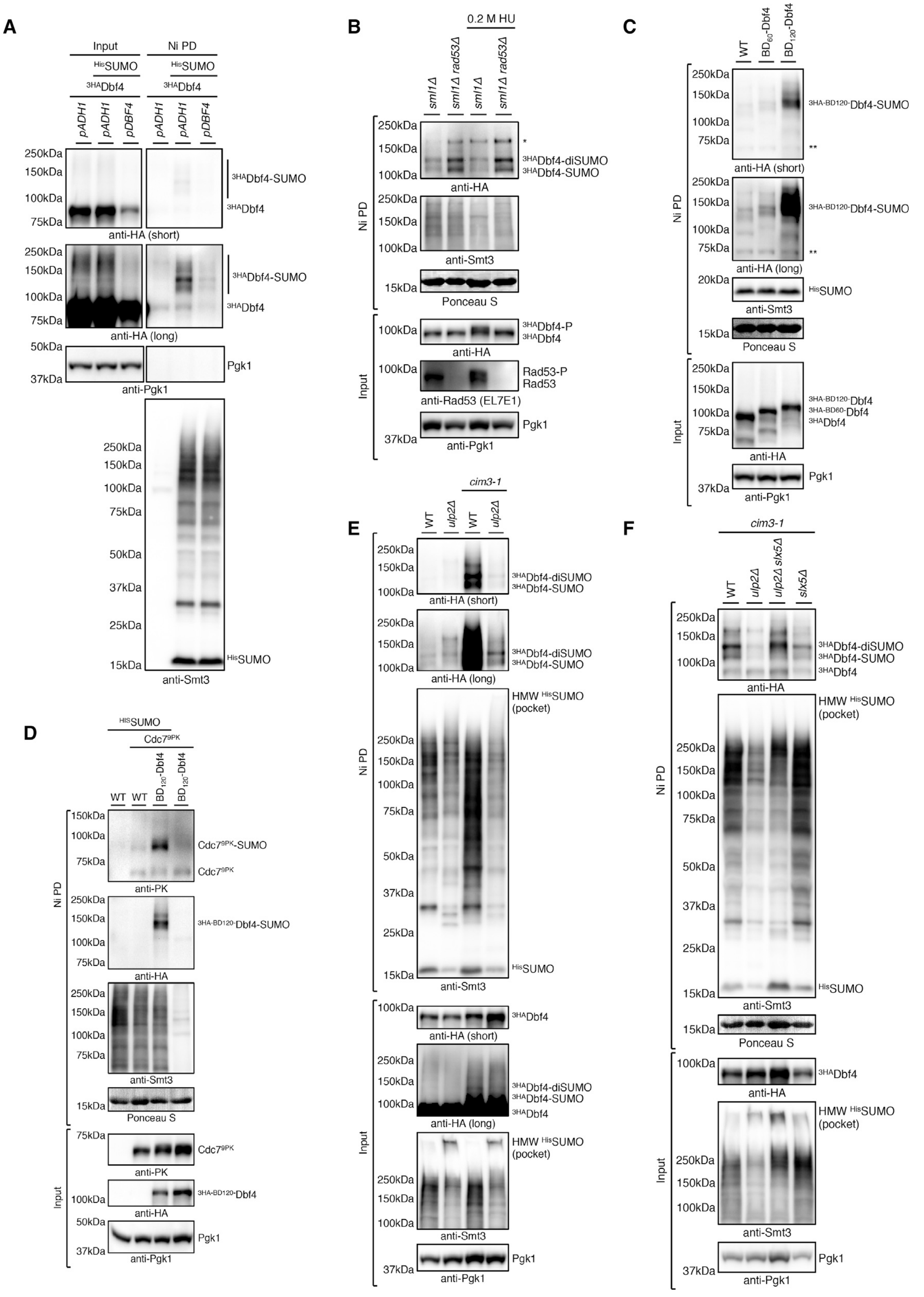

(legend on next page)

*slx5*Δ cells (Figure 3F), suggesting that Ulp2 protects mono- and/or multiSUMOylated DDK against Slx5/8 STUbL-mediated turnover.

SUMO Chains Target DDK for Slx5/8 STUbL-Mediated Proteasomal Degradation

We next asked whether any of the three known SUMO ligases operating in mitotic yeast cells—Siz1, Siz2, and Mms21—mediate DDK SUMOylation. ^{His}SUMO Ni PD revealed that *siz1*Δ and *siz2*Δ mutants, but not the *mms21-11* mutant defective in its SUMO ligase activity, had lower levels of SUMOylated Cdc7 and Dbf4 compared with the WT (Figures 4A and S4A). Thus, DDK is being SUMOylated by Siz1 and Siz2, both of which harbor DNA-binding SAF-A/B, Acinus, PIAS (SAP) domains (Jentsch and Psakhye, 2013).

We next investigated whether SUMO chains are indeed the signal for the STUbL-mediated proteasomal degradation of DDK. We performed Ni PD in *cim3-1* cells expressing, as a single source of SUMO, either ^{His}SUMO or a His-tagged SUMO variant in which all lysine residues were mutated to arginine (*KRall*), preventing formation of lysine-linked SUMO chains. Expressing the *KRall* SUMO mutant abolished the accumulation of HMW ^{His}SUMO conjugates in *ulp2*Δ cells, increased the abundance of mono- and/or multiSUMOylated Dbf4 and Cdc7 species in the WT, and suppressed their instability in *ulp2*Δ cells (Figures 4B and S4B). Thus, SUMO chains target replication-engaged SUMOylated DDK for Slx5/8 STUbL-mediated proteasomal degradation in the absence of Ulp2.

The Cdc48/p97 ATPase emerged as an important regulator of SUMO and ubiquitin conjugates on chromatin, often in collaboration with STUbLs (Bergink et al., 2013; Køhler et al., 2015). First, we found Y2H interaction between Dbf4 and the Cdc48 substrate-recruiting co-factor Ufd1 (Figure S4C). Next, using the temperature-sensitive *cdc48-6* and *cdc48-3* mutants, we observed an accumulation of SUMOylated Dbf4 species following Cdc48 inactivation (Figures 4C and S4D). Thus, Cdc48 segregase assists with turnover of SUMOylated DDK.

Further confirming the uncovered links between components of the SUMO and ubiquitin pathways and DDK, we observed binding of Dbf4 to the SUMO ligase Siz2 and the Slx5 subunit of the Slx5/8 STUbL by Y2H (Figure 4D). Moreover, Y2H revealed binding of Ulp2 to Cdc48, which was dependent on the N terminus (amino acids [aa] 1–400) of Ulp2. Similarly, N-terminally truncated, catalytically inactive Ulp2 ($Ulp2_{CD}$-N_{400}) failed to interact with Dbf4 in both orientations (Figure 4D), indicating that Ulp2 uses certain motif(s) at its N terminus for binding to its SUMOylated substrates. Supporting this notion, we observed loss of Dbf4 binding to Ulp2 but also to Siz2 and Slx5 when both SUMO ligases, Siz1 and Siz2, responsible for SUMOylation of DDK were deleted in the Y2H reporter strain (Figure 4E). Dbf4 binding to Siz2 thus seems to be Siz1-dependent. The presence of Siz2, in turn, is largely required for the interaction between Dbf4 and both Slx5 and Ulp2 and also between Cdc48 and Ulp2 (Figure S4E).

N-Terminal SUMO-Interacting Motifs of Ulp2 Mediate Binding to and Protection of SUMOylated DDK

Regarding how Ulp2 is recruited to mono- and/or multiSUMOylated DDK that it protects, we found that artificial targeting of DDK to Gal4-binding sites using the BD_{120}-Dbf4 fusion (Figures 3C and 3D) causes concomitant recruitment of Ulp2 specifically to the genes of the yeast galactose regulon (Figure S5A). Thus, SUMOylated DDK is capable of directly recruiting Ulp2 to chromatin even when DDK is not in the context of the replisome, suggesting how Ulp2 might become enriched at replication origins.

Next, following the observed loss in binding of Dbf4 to the truncated variant $Ulp2_{CD}$-N_{400} in Y2H (Figure 4D), we examined whether this region contains putative SIMs, short hydrophobic sequences that mediate binding to SUMO (Song et al., 2004). Interestingly, we found potential SIMs in Ulp2 that strongly resemble validated N-terminal SIMs of the Slx5 STUbL subunit (Xie et al., 2010), both in number and relative positioning (Figure 5A). When we abolished either the first three (*ulp2-sim1,2,3*) or all five (*ulp2-sim1,2,3,4,5*) potential N-terminal SIMs in Ulp2 by replacing hydrophobic residues with alanines, the interaction of Dbf4 with Ulp2 in Y2H was lost, similar to the N-terminally truncated variant $Ulp2_{CD}$-N_{400} (Figure 5B). We next recombinantly expressed the N terminus of Ulp2 as glutathione S-transferase (GST) fusion (GST-$Ulp2_{1-400}$) and found that it indeed interacts with both recombinant free yeast Smt3 (^{His}SUMO) and human poly-SUMO3 chains *in vitro* (Figures 5C and 5D). Strikingly, the N terminus of Ulp2 shows a strong preference for binding poly-SUMO3 chains longer than 5 SUMO3 units (Figure 5D), in line with the idea that Ulp2 recognizes its SUMO-chain-modified substrates via its five N-terminal SIMs. To address whether the identified putative SIMs are important for SUMO binding, we recombinantly expressed the GST-$Ulp2_{1–400}$ fragments with *sim1,2,3* and *sim1,2,3,4,5* mutations. These variants, when expressed in *E. coli*, showed more degradation products that

Figure 3. Chromatin-Bound DDK Engaged in Replication Is SUMOylated and Protected by Ulp2 against Slx5/8 STUbL-Mediated Proteasomal Degradation

(A) Dbf4 is SUMOylated. Shown is denaturing Ni-NTA pull-down (Ni PD) of ^{His}SUMO conjugates from cells expressing ^{3HA}Dbf4 under the control of an endogenous (*pDBF4*) or strong constitutive *ADH1* promoter (*pADH1*).

(B) Dbf4 SUMOylation is increased in the absence of the Rad53 checkpoint. Shown is ^{His}SUMO Ni PD from *sml1*Δ and *sml1*Δ *rad53*Δ cells or untreated cells grown to an optical density of a sample at 600 nanometers (OD_{600}) of 0.7 and then shifted to 0.2 M HU for 90 min. Ni PD efficiency was assayed using an anti-Smt3 antibody and staining with Ponceau S. Asterisks denote cross-reactivity of the anti-HA antibody.

(C) Artificial targeting of Dbf4 to DNA triggers its SUMOylation; as in (B), but with WT cells expressing either ^{3HA}Dbf4 or its BD_{60} and BD_{120} fusions.

(D) Artificial DNA targeting of Dbf4 triggers SUMOylation of the DDK catalytic subunit Cdc7; as in (C), but with cells expressing either untagged Cdc7 or $Cdc7^{9PK}$.

(E) SUMOylated Dbf4 species accumulate in the *cim3-1* proteasome-defective mutant but not in *cim3-1 ulp2*Δ; as in (B), but with WT, *ulp2*Δ, *cim3-1*, and *cim3-1 ulp2*Δ cells.

(F) Decreased levels of SUMOylated Dbf4 in *cim3-1 ulp2*Δ cells are restored in the *cim3-1 ulp2*Δ *slx5*Δ mutant; as in (E), but with *cim3-1*, *cim3-1 ulp2*Δ, *cim3-1 slx5*Δ, and *cim3-1 ulp2*Δ *slx5*Δ cells.

See also Figure S3.

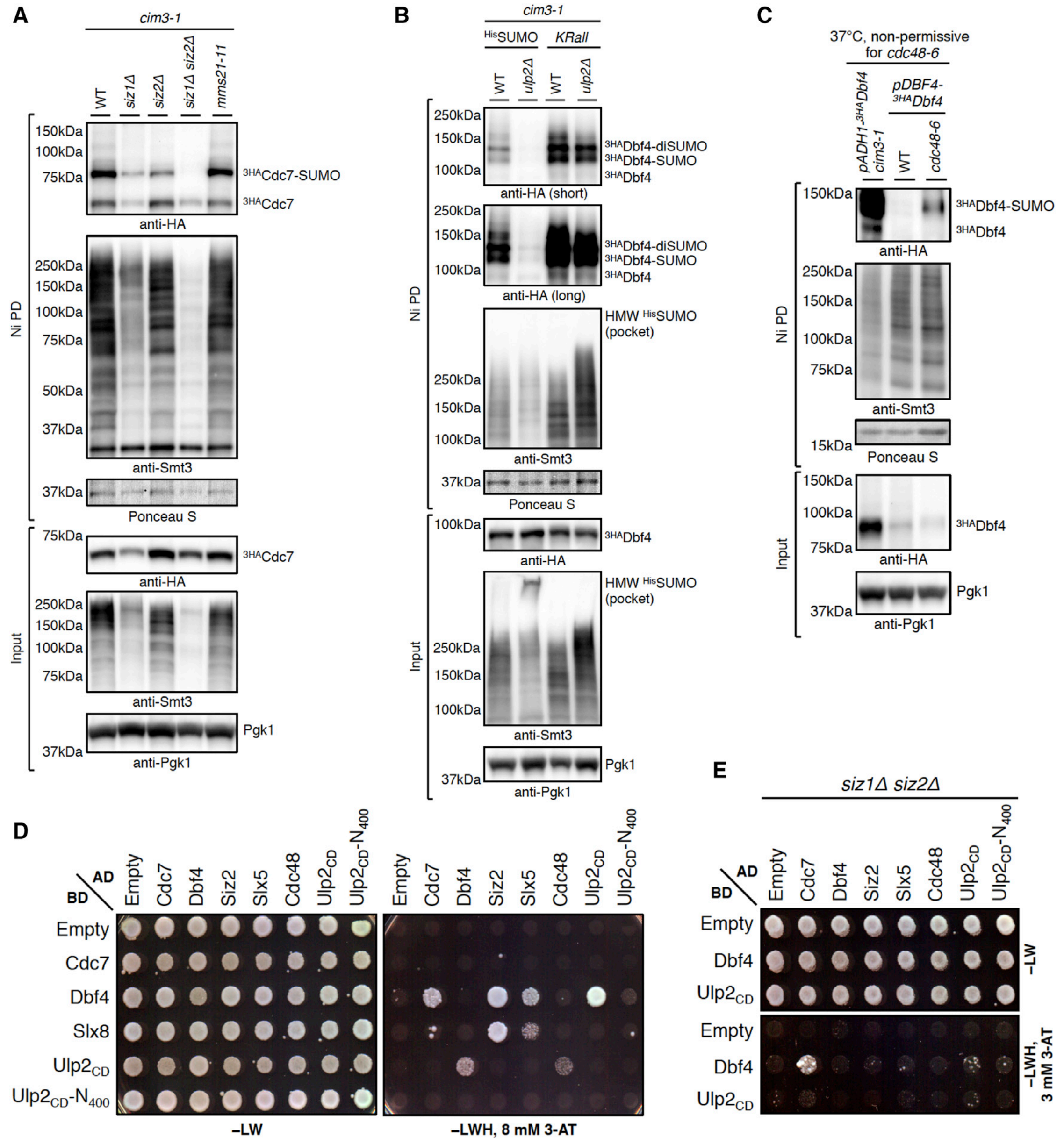

Figure 4. Ulp2 Counteracts Siz1/2-Mediated SUMO Chain Formation, which Targets SUMOylated DDK for Cdc48 ATPase-Assisted Proteasomal Degradation

(A) SUMOylation of Cdc7 is mediated by the SUMO ligases Siz1 and Siz2. Shown is ^{His}SUMO Ni PD from the *cim3-1* mutant expressing ^{3HA}Cdc7 (WT) and cells additionally lacking the SUMO ligase Siz1, Siz2, or both or carrying the *mms21-11* allele.

(B) The decreased levels of SUMOylated Dbf4 species in *cim3-1 ulp2*Δ are restored when, instead of ^{His}SUMO, a lysine-less SUMO variant (*KRall*) is expressed.

(C) ^{His}SUMO Ni PD from WT cells and a temperature-sensitive *cdc48-6* mutant expressing ^{3HA}Dbf4 under the control of an endogenous promoter (*pDBF4*), grown to an OD_{600} of 0.7 at 28°C and then shifted to 37°C for 3 h. SUMOylated Dbf4 species accumulate in the *cdc48-6* mutant compared with WT cells.

(D) Dbf4 interacts in Y2H with Siz2, Slx5, and Ulp2 (catalytically dead Ulp2-C624S; $Ulp2_{CD}$) but not with its N-terminally truncated variant $Ulp2_{CD}$-N_{400}. Like Dbf4, Cdc48 interacts with Ulp2 depending on its N terminus. 8 mM 3-amino-triazole (3-AT) was added to reduce auto-activation of the *HIS3* reporter gene.

(E) Interaction of Dbf4 with Siz2, Slx5, and Ulp2 is lost in the absence of Siz1 and Siz2. Dbf4 binding to Siz2 is Siz1-dependent.

See also Figure S4.

also retained the ability to bind free SUMO (Figure S5B). Importantly, however, when adjusted for the overall GST signal, the GST-$Ulp2_{1\text{-}400}$ fusions with SIMs mutated showed strongly reduced (almost to the background level in the case of *sim1,2,3,4,5)* binding to SUMO compared with WT GST-$Ulp2_{1–400}$ (Figure S5C).

In vivo, *ulp2-sim* mutants phenotypically resembled *ulp2*Δ cells regarding slow growth and temperature sensitivity.

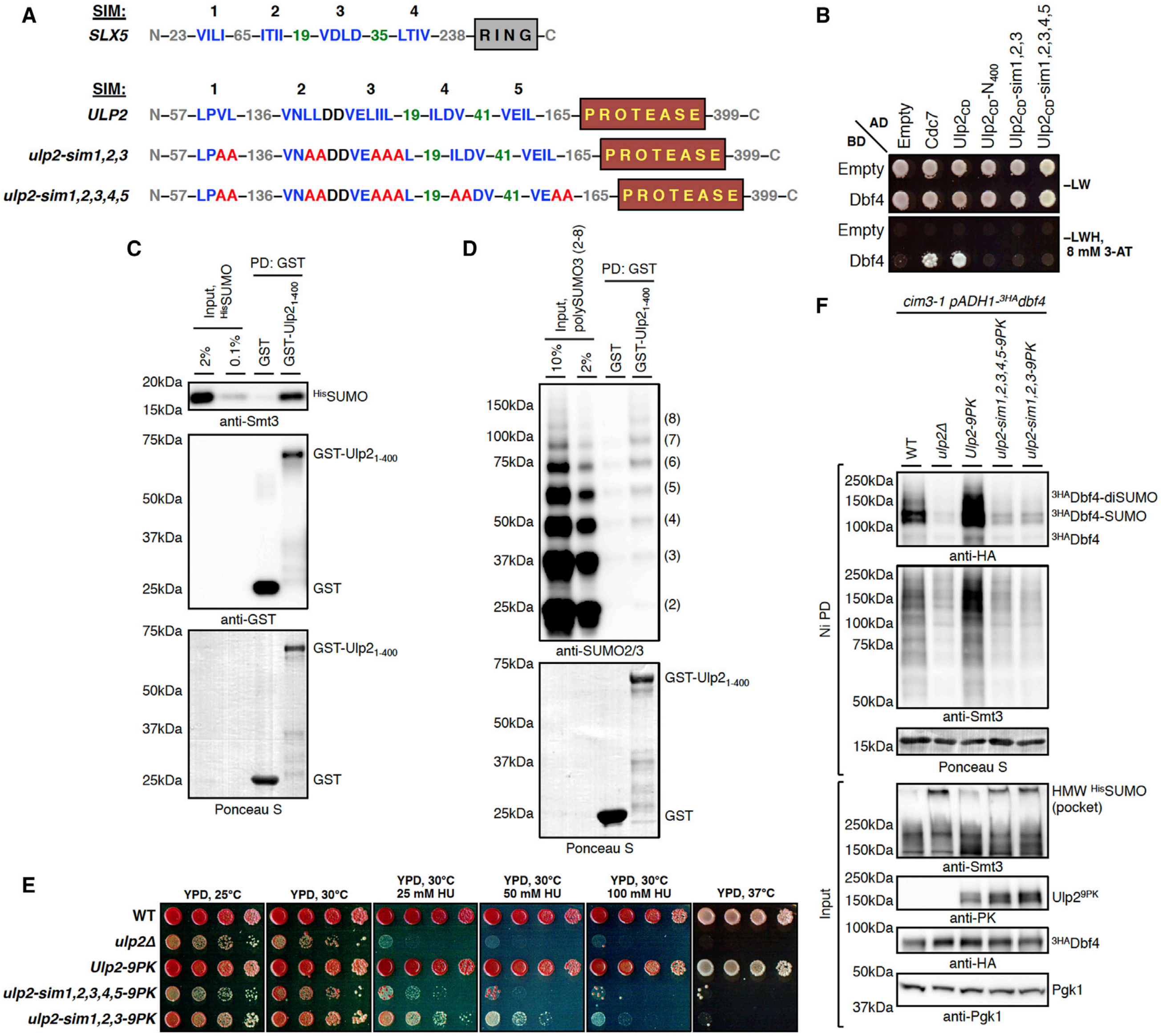

Figure 5. SUMO-Interacting Motifs at the N terminus of Ulp2 Mediate Binding to Dbf4 and Are Required to Protect SUMOylated DDK from Proteasomal Turnover

(A) Predicted SIMs in the Ulp2 N terminus resemble confirmed Slx5 SIMs in number and relative positioning (highlighted green). Shown is a schematic representation of Slx5 and Ulp2 with the RING and protease domains, SIMs (blue), and introduced mutations (red) that disrupt potential SIMs.

(B) Y2H interaction of Dbf4 with Ulp2 is abolished when either first three or all five N-terminal putative SIMs of Ulp2 are mutated, similar to $Ulp2_{CD}$-N_{400}.

(C and D) Recombinant GST fusion of the Ulp2 N terminus (aa 1–400) binds both yeast-free SUMO (C) and human poly-SUMO3 chains (D) *in vitro*, with a stronger preference for chains having more than 5 SUMO3 moieties.

(E) *ulp2-sim* mutants phenotypically resemble *ulp2*Δ cells regarding slow growth and temperature sensitivity but have lower sensitivity to HU.

(F) Both *ulp2-sim* mutants similar to *ulp2*Δ fail to protect SUMOylated Dbf4 against SUMO chain/STUbL-mediated proteasomal degradation. Shown is ^{His}SUMO Ni PD from *cim3-1*, *cim3-1 ulp2*Δ, and *cim3-1* cells carrying either $Ulp2^{9PK}$ or its SIM mutant variants.

See also Figure S5.

However, both *ulp2-sim* mutants showed lower sensitivity to HU compared with *ulp2*Δ, and *ulp2-sim1,2,3* had milder phenotypes compared with *ulp2-sim1,2,3,4,5*, suggestive of residual protease activity toward SUMO conjugates (Figure 5E). Finally, we asked whether the identified N-terminal SIMs of Ulp2 are required for protection of mono- and/or multiSUMOylated DDK against STUbL-mediated proteasomal turnover. Both *ulp2-sim* mutants failed to protect mono- and/or multiSUMOylated Dbf4 (Figure 5F) and Cdc7 (Figure S5D) against proteasomal degradation and accumulated HMW ^{His}SUMO conjugates, similar to the *ulp2*Δ mutant, despite being expressed at WT levels. Thus, Ulp2 protects replication-engaged mono- and/or multiSUMOylated

DDK against Slx5/8-mediated proteasomal degradation by binding to growing SUMO chains via five SIMs located in its N terminus and trimming them from the distal ends.

Ulp2 Safeguards SUMOylated DDK, Allowing Replication Initiation

We next addressed what the functional consequence would be for cells of losing the Ulp2-mediated protection of the SUMOylated DDK pool engaged in replication. First, using a genetic approach, we found that *ulp2*Δ has synergistic growth defects with temperature-sensitive mutations in DDK, such as *dbf4-1*, *cdc7-4*, and *cdc7-1*, at temperatures permissive for the single mutants (Figures 6A, 6B, and S6A), suggesting that, in the absence of Ulp2, DDK function is further compromised in these mutants. Importantly, the above-mentioned synthetic lethality of *dbf4-1 ulp2*Δ, *cdc7-4 ulp2*Δ, and *cdc7-1 ulp2*Δ cells is rescued by expression of the *smt3-KRall* SUMO variant, which cannot form lysine-linked SUMO chains, as a single source of SUMO (Figures 6A, 6B, and S6A).

We then asked whether the function of Ulp2 in supporting early stages of replication (Figure 2) converges on the ability and/or availability of DDK to mediate Mcm4 phosphorylation, a modification required to initiate DNA replication (Bell and Labib, 2016; Randell et al., 2010). To this end, we performed IP of $Mcm4^{13MYC}$ and detected DDK-phosphorylated Mcm4 species using a phosphospecific antibody that recognizes phosphorylation of Mcm4-S82-D83, an intrinsic DDK target site (Randell et al., 2010). Importantly, we found a reduction in DDK-mediated Mcm4 phosphorylation in *ulp2*Δ cells and suppression of this defect by the *slx5*Δ mutation (Figure 6C). We note that the signal for Mcm4 phosphorylation is higher in WT compared with *slx5*Δ and *ulp2*Δ *slx5*Δ cells, suggesting that Slx5 might positively influence DDK activity by targeting other substrates that, at the moment, remain unknown. We ascertained that the detected Mcm4 represents phosphorylated species by subjecting the IP material from WT cells to lambda phosphatase (λ) treatment in the absence or presence of phosphatase inhibitors (Inh). Thus, Ulp2 allows efficient Mcm4 phosphorylation.

To unambiguously link the unscheduled Slx5/8 STUbL-mediated proteasomal degradation of mono- and/or multiSUMOylated DDK to the replication defects observed in *ulp2*Δ cells, we sought to map the SUMO acceptor lysine residues on both Dbf4 and Cdc7 DDK subunits. Using the *cim3-1* background and a systematic mutagenesis approach to replace lysines (K) with arginines (R) in full-length 3HA-tagged Dbf4, we found, via Ni PD, that mutations in K6, K14, and K432 strongly reduced Dbf4 SUMOylation, with K432 being the major SUMO acceptor site (Figure S6B). In parallel, we analyzed the published structure of the human DDK (Hughes et al., 2012) for exposed lysine residues conserved from yeast to humans that may lie within a SUMO consensus motif, ψ-K-x-E/D (ψ, a hydrophobic amino acid; x, any amino acid). We found that K16 in budding yeast Cdc7, corresponding to K41 in human Cdc7, meets these requirements and is a major SUMOylation site (Figure S6C). K34 also contributes to Cdc7 SUMOylation to a minor extent (Figure S6C), in addition to several other lysines, similar to the situation in human cells, where, using a high-throughput mass spectrometry approach, multiple SUMOylation sites have been mapped in DDK (see the list in Figure S6C; Hendriks et al., 2017; Lamoliatte et al., 2014). Thus, DDK SUMOylation is conserved from yeast to humans and targets a conserved site in Cdc7.

We next constructed a SUMOylation-defective *ddk-KR* mutant with mutations in the major Dbf4 and Cdc7 SUMOylation sites (*cdc7-K16R dbf4-K6R*, *K14R*, *K432R*) that does not need Ulp2 for protection. Importantly, the defect in DDK-mediated Mcm4 phosphorylation observed in *ulp2*Δ cells was rescued by the *ddk-KR* mutation, and the levels of phosphorylated Mcm4 were slightly increased in *ddk-KR* (Figure 6D). Moreover, the replication onset defect observed with BrdU incorporation in *ulp2*Δ cells was suppressed by *ddk-KR*, similar to *slx5*Δ (Figure 6E). Neither *ddk-KR* nor *slx5*Δ altered the replication origin usage profile. Finally, the slower S phase progression of *ulp2*Δ cells in unperturbed conditions was suppressed by the *ddk-KR* mutation (Figure 6F, 50–60 min), as it was by the *smt3-KRall* mutation (Figure S6D, 45–60 min). Furthermore, we observed similar suppression of the replication initiation defect in *ulp2*Δ cells by the *ddk-KR* mutation under conditions that allow yeast cells to replicate DNA in G1 using the S-phase cyclin-dependent kinase (S-CDK) bypass setting (Figure S6E). In S-CDK bypass cells, the essential function of S-CDK in replication initiation is being overcome by combining *sld3-dpb11* fusion with galactose-induced overexpression of a phosphomimetic *sld2-T84D* mutation (Zegerman and Diffley, 2007). Under such conditions, DDK becomes limiting for DNA replication, and ectopic overexpression of *DBF4* is necessary for extensive DNA synthesis in α-factor-arrested G1 cells (Zegerman and Diffley, 2007). In this S-CDK bypass system, the *ulp2*Δ mutation caused substantial delays in replication, whereas the *ddk-KR* mutation rescued this defect (Figure S6E). Moreover, *ddk-KR* alone allowed a similar extent of DNA replication in G1 as *DBF4* overexpression or replacement of the chromosomal *MCM4* with $mcm4^{\Delta 74-174}$ (Figure S6E), an allele known to bypass the DDK requirement for replication initiation by alleviating inhibitory activity in Mcm4 (Sheu and Stillman, 2010). These results indicate that Ulp2 facilitates replication onset by preventing unscheduled SUMO-chain-targeted Slx5/Slx8 STUbL-mediated degradation of mono- and/or multiSUMOylated DDK engaged in replication.

Slx5/8 Substrate Trapping and SILAC Screens Identify MCM Subunits and Other Replication Factors as Potential Targets of Ulp2 and Slx5/Slx8

Our current knowledge of substrates regulated by the interplay between SUMO chains, Ulp2 protease, and Slx5/8 STUbL is very limited, and therefore we aimed to identify other potential targets of the regulatory timing mechanism uncovered here for DDK. To detect degradation-prone SUMOylated targets of the Slx5/8 STUbL, we decided to employ the ubiquitin ligase substrate-trapping approach (Mark et al., 2014), which is based on fusion of the UBA domains derived from the Rad23 and Dsk2 ubiquitin receptors to the ubiquitin ligase under investigation, here Slx5/8 (Figure S7A), in *ulp2*Δ *cim3-1* ^{His}*SUMO* cells. Generated Slx8-UBA-domain fusions should bind ubiquitylated proteins in the vicinity of the Slx5/8 ubiquitin ligase, including its own substrates, when the ligase is catalytically active but not

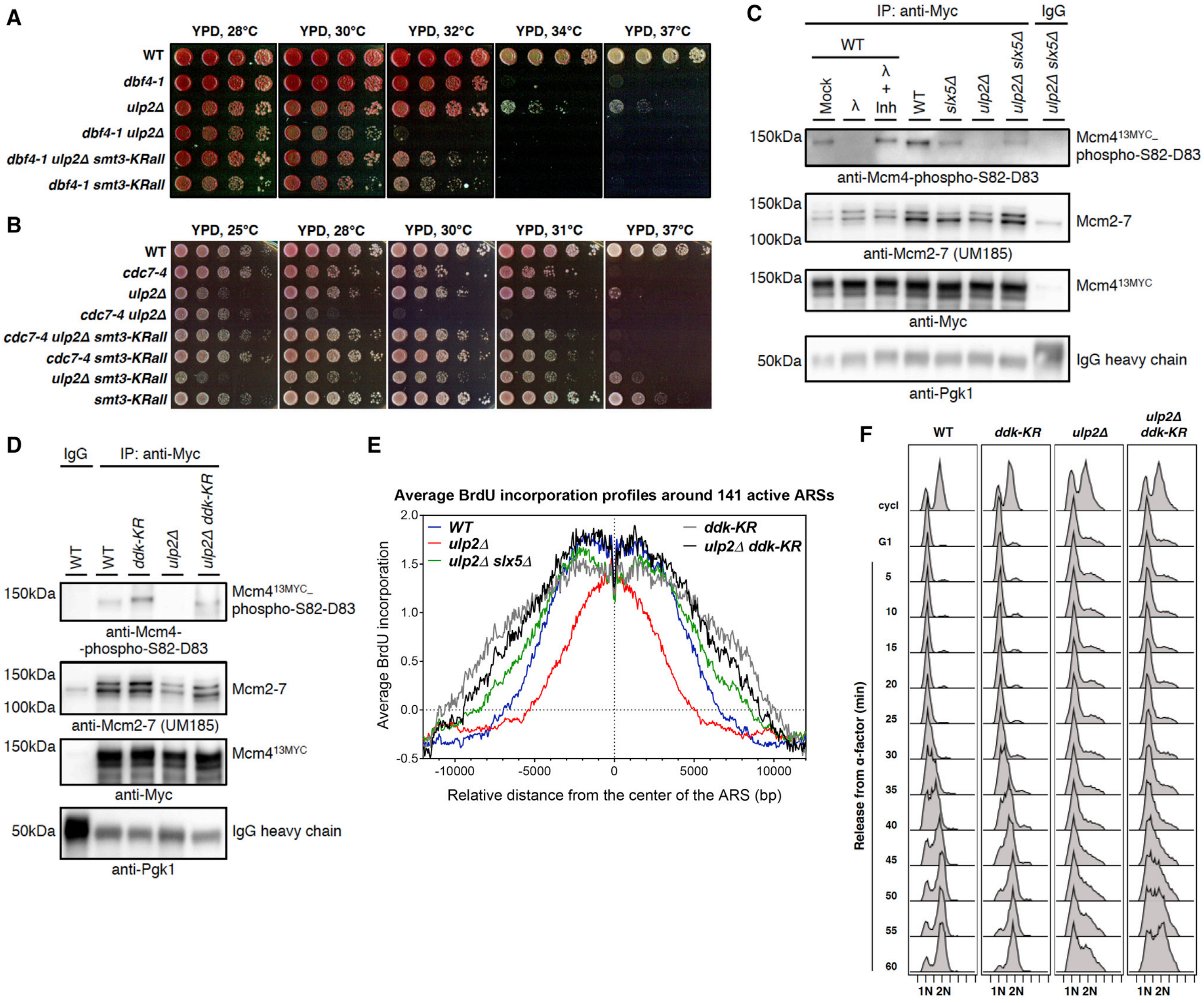

Figure 6. Ulp2 Supports Efficient Replication Onset by Safeguarding MonoSUMOylated DDK Engaged in Replication

(A and B) Synthetic lethality of *dbf4-1 ulp2*Δ and *cdc7-4 ulp2*Δ cells at permissive temperatures for *dbf4-1* and *cdc7-4* single mutants is suppressed by a lysine-less SUMO variant (*smt3-KRall*).

(C) Reduced DDK-mediated Mcm4 phosphorylation in *ulp2*Δ cells is suppressed by deleting *SLX5*. Shown is IP of Mcm4^{13MYC} from exponentially growing WT, *slx5*Δ, *ulp2*Δ, and *ulp2*Δ *slx5*Δ cells. The specificity of the anti-Mcm4-phospho-S82-D83 antibody was evaluated by lambda phosphatase treatment (λ) with or without phosphatase inhibitors (Inh).

(D) The SUMOylation-defective *ddk-KR* mutant rescues reduced DDK-dependent Mcm4 phosphorylation in *ulp2*Δ cells; as in (C), but Mcm4^{13MYC} IP from WT, *ddk-KR*, *ulp2*Δ, and *ulp2*Δ *ddk-KR* cells.

(E) The decrease in BrdU incorporation in *ulp2*Δ cells is suppressed by *ddk-KR*, similar to *slx5*Δ. Shown is BrdU IP-on-chip analysis of cells released into S phase in the presence of 0.2 M HU and BrdU for 90 min after G1 arrest.

(F) The S phase progression defect in *ulp2*Δ cells is suppressed by *ddk-KR*. Exponentially growing WT, *ddk-KR*, *ulp2*Δ, and *ulp2*Δ *ddk-KR* cells (cycl) arrested in G1 phase by α-factor were released into yeast extract-peptone-dextrose (YPD) media at 25°C, and samples were taken every 5 min for FACS.

See also Figure S6.

when it is inactivated by point mutations in the RING domain of Slx5 (Figures 7A and S7B). Because both subunits of Slx5/Slx8-UBA additionally carry hemagglutinin (HA) tags, trapped ubiquitylated proteins can be co-immunoprecipitated together with other specific and unspecific Slx5/8 interactors in the first step of anti-HA IP and identified using mass spectrometry (Figure S7C). Because in most cases Slx5/8 targets substrates are marked by SUMO modifications, SUMOylated proteins are expected to be enriched by subsequent (second step) Ni PD of ^{His}SUMO conjugates when Slx5/8 is catalytically active but not when it is inactivated and can only trap Slx5/8-independent ubiquitylated proteins, serving as a good indicator of the substrate-trapping specificity. It was indeed the case, as judged by western blot controls of the ligase substrate trapping with

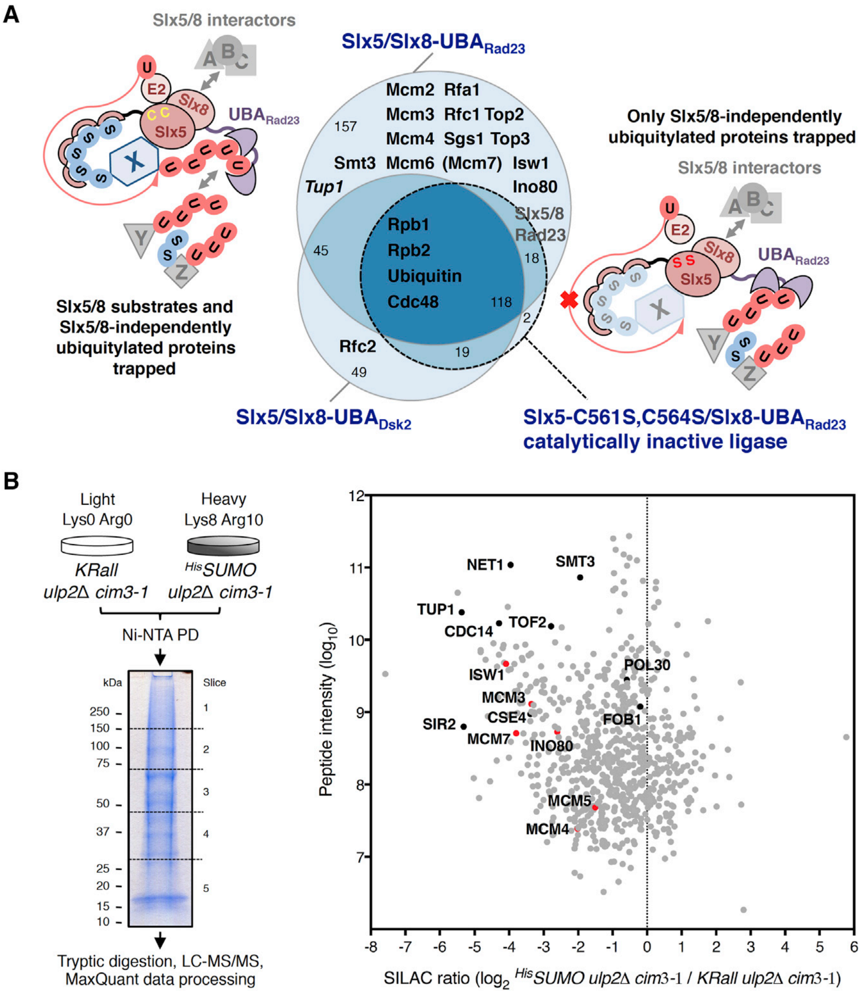

Figure 7. MCM Helicase and Other Replication Factors Are Potential Substrates of Ulp2 Protease and Slx5/8 STUbL

(A) Slx5/Slx8-UBA STUbL substrate trapping from *cim3-1 ulp2*Δ cells. The Euler diagram shows proteins detected following IP with the indicated Slx5/Slx8-UBA substrate traps. Proteins (Table S1) were identified using Scaffold with stringent criteria. Mcm7 (in parentheses) was detected with one unique peptide. Tup1 (in italics) is a validated SUMOylated Slx5/8 substrate.

(B) Outline of the SILAC experiment aiming to detect degradation-prone SUMOylated substrates that decrease in abundance in a SUMO-chain-dependent manner in *ulp2*Δ *cim3-1* cells (left). SILAC ratios for 726 quantified proteins were plotted against the sum of the relevant peptide intensities (right). MCM subunits and replication factors detected by Slx5/8 ligase substrate trapping in (A) are colored red.

See also Figure S7.

catalytically active stable Slx5/Slx8-UBA$_{Rad23}$, highly unstable Slx5/Slx8-UBA$_{Dsk2}$, and catalytically inactive stable Slx5-C561S,C564S/Slx8-UBA$_{Rad23}$ substrate traps (Figure S7D, samples 4–6). Interestingly, the utilized approach led to specific identification of the MCM helicase subunits and other replication factors that have been shown previously to be targeted by SUMO modification (Cremona et al., 2012; Wei and Zhao, 2016) as potential substrates of the Slx5/8 STUbL (Figure 7A; Table S1). Significantly fewer hits were identified following the second step Ni PD of ^{His}SUMO conjugates (Figure S7E), likely because of low protein amounts isolated, nevertheless confirming some of the potential Slx5/8 substrates from the first step, such as Sgs1 and Top2.

We aimed to complement the aforementioned findings by searching for degradation-prone SUMO conjugates that decrease in abundance in the absence of Ulp2 specifically in a SUMO-chain-dependent manner, as is the case for DDK (Figures 4B and S4B). Therefore, we compared, by ^{His}SUMO Ni PD using a SILAC-based mass spectrometry approach (Mann, 2006), the levels of SUMO conjugates in *ulp2*Δ *cim3-1* mutants expressing either the WT or the *KRall* SUMO variant (Figure 7B, left). This screen quantified 726 potential SUMO conjugates (Figure 7B, right); the abundance of most of them did not change significantly (e.g., proliferating cell nuclear antigen [PCNA] or Pol30 in yeast), whereas SUMO conjugates pulled down from the *KRall* mutant were more abundant in general (Smt3; Figure 4B, Ni PD). Strikingly, among the SUMO substrates strongly enriched in the sample derived from SUMO-chainless *ulp2*Δ cells were, again, MCM subunits and other replication factors (Figure 7B, right) identified earlier by Slx5/8 ligase substrate trapping (Figure 7A). Importantly, we also found the known SUMOylated Ulp2 substrates Net1, Tof2, Cdc14, and Tup1 (Liang et al., 2017) and the confirmed Slx5/8 targets Cse4, Tof2, and Tup1 (Liang et al., 2017; Ohkuni et al., 2016; Wang et al., 2006) to decrease in abundance in *ulp2*Δ cells depending on SUMO chains. The presence of these previously reported hits and overlap between the two screens highlight the MCM helicase as a likely substrate of SUMO-chain-targeted Slx5/Slx8 STUbL-mediated degradation regulated by Ulp2. Because MCM is extracted and degraded during replication termination in an SCF(Dia2)-mediated manner (Maric et al., 2014), and *slx5*Δ and *dia2*Δ mutations are synthetic lethal (Figure S7F; Blake et al., 2006), it is possible that SUMO-chain- and Slx5/8-mediated degradation might operate during replication termination as a backup pathway for SCF(Dia2)-mediated replisome disassembly, which targets Mcm7. In all, we propose SUMO-chain/Ulp2-protease-regulated proteasomal degradation to act as a mechanism that times the availability of functionally engaged SUMO-modified protein pools during replication.

DISCUSSION

Here we uncovered that the SUMO protease Ulp2, controlling the physiological extent of SUMO conjugation, serves as a

guardian against unscheduled SUMO chain assembly on DDK, timing its proteasomal degradation. This action of Ulp2, likely also involving MCM subunits, allows replication initiation and may have other functions beyond replication by means of other substrates. We propose that SUMO chains regulated by the Ulp2 protease function like a countdown timer when they are assembled on the substrates of STUbLs, which may channel them either for degradation or equip them with other functional properties of the ubiquitin code.

Our findings indicate that Ulp2 interacts and cooperates with DDK to support replication onset. DDK becomes SUMOylated with single SUMO moieties (monoSUMOylation) at multiple sites (multiSUMOylation) when the kinase engages in replication and is bound to chromatin. However, monoSUMOylation can be extended to SUMO chains, which can recruit STUbLs and mediate proteasomal degradation of the modified substrate. Indeed, we find that specifically SUMO chains promote Slx5/8 STUbL-mediated and Cdc48 segregase-assisted proteasomal degradation of SUMOylated DDK when not protected by Ulp2. Loss of Ulp2 causes defects in MCM activation and replication onset. Notably, these replication defects are suppressed by a SUMOylation-defective *ddk-KR* mutant that no longer requires Ulp2 for protection against Slx5/8 STUbL. Moreover, *ddk-KR* also allows yeast cells to replicate DNA under the S-CDK bypass condition, when DDK becomes limiting for DNA replication in G1-arrested cells (Zegerman and Diffley, 2007), because of APC/C-Cdc20-mediated proteasomal destruction of Dbf4 in M/G1 (Cheng et al., 1999; Ferreira et al., 2000). Thus, Ulp2 facilitates replication onset by preventing unscheduled SUMO-chain-targeted Slx5/8 STUbL-mediated degradation of the mono- and/or multiSUMOylated DDK pool engaged in replication.

We note that the SUMO-chain/Ulp2 protease-mediated timing of proteasomal degradation uncovered here is fundamentally different in significance from the previously described APC/C-mediated Dbf4 destruction mechanism. Although APC/C-mediated degradation limits Dbf4 protein levels in M/G1 to prevent unscheduled replication (Cheng et al., 1999; Ferreira et al., 2000), Ulp2 engages and stabilizes mono- and/or multiSUMOylated DDK functionally engaged in replication, allowing efficient replication onset. At the same time, SUMOylation marks the active DDK pool for subsequent SUMO-chain-targeted proteasomal turnover, potentially to prevent re-replication and to facilitate replication termination at a time when Ulp2 is no longer concentrated in the proximity of DDK or becomes inhibited by the Cdc5 kinase in mitosis (Baldwin et al., 2009).

SUMOylation has been proposed to happen in waves that affect functional protein groups (Jentsch and Psakhye, 2013). In the context of double-strand break repair, protein group SUMOylation fosters interactions between multiple homologous recombination factors, making the repair process more efficient (Psakhye and Jentsch, 2012). Similar SUMOylation waves are envisaged to happen in other cellular settings, one of which may be replication initiation. Indeed, many replisome components loaded at origins of replication, including – origin recognition complex (ORC) and MCM, are SUMOylated (Cremona et al., 2012; Golebiowski et al., 2009; Wei and Zhao, 2016). Here, using two proteomic approaches, we identify MCM subunits and several replication factors along with other previously reported groups, such as the ones implicated in ribosomal DNA silencing (Liang et al., 2017), as potential substrates of both Ulp2 and Slx5/8, channeled for proteasome-mediated degradation in a SUMO-chain-dependent fashion. Thus, enrichment of Ulp2 at origins of replication is likely coupled with the wave of SUMOylation occurring during replication initiation to preserve replication-engaged factors that can be recognized by STUbLs until their turnover is scheduled. The MCM helicase has been found to be extracted from chromatin by the Cdc48 segregase during replication termination upon its ubiquitylation by the SCF(Dia2) ubiquitin ligase (Maric et al., 2014; Moreno et al., 2014) but also appears to be degraded in a Slx5/8- and SUMO-chain-dependent manner. Because mutations in Dia2 are synthetic lethal with loss of Slx5/8, it is possible that the SCF(Dia2) and Slx5/8 SUMO chain pathways act in compensation to promote degradation of replication proteins, such as MCM, before activation of anaphase-promoting complex/cyclosome (APC/C) in mitosis.

How exactly Slx5/8 STUbL recognizes its targets is not well understood, but because all SUMOylated factors can potentially undergo SUMO chain assembly, whereas only a few Slx5/8 substrates are known, it is likely that a specific degradation signal (degron) is additionally present in polySUMOylated substrates that undergo Slx5/8-dependent turnover. Moreover, Ulp2 and Slx5/8 STUbL seem to share several substrates. From this perspective, our identification of DDK as a substrate of both Ulp2 and Slx5/8 makes it a good study case for future mapping of the Slx5/8 degron and the Ulp2 recruitment motif.

In conclusion, our work provides mechanistic insights into how dynamic SUMO modification of DDK and other replication factors at replication origins cooperate with the SUMO-chain-editing protease Ulp2 *in vivo* to allow chromosome replication onset, highlighting an important function of SUMO chain signaling in the replication initiation context and opening ways to address other unsolved puzzles in the SUMO pathway.

STAR★METHODS

Detailed methods are provided in the online version of this paper and include the following:

- KEY RESOURCES TABLE
- LEAD CONTACT AND MATERIALS AVAILABILITY
- EXPERIMENTAL MODEL AND SUBJECT DETAILS
  - Yeast Strains
- METHOD DETAILS
  - Yeast Techniques
  - TCA Protein Precipitation
  - Ni-NTA Pull-down of ^{His}SUMO Conjugates
  - Immunoprecipitation and Phosphatase Treatment
  - GST *in vitro* pull-down assays
  - ChIP, ChIP-on-chip, BrdU IP-on-chip
  - ChIP-qPCR
  - 2D Gel Electrophoresis
  - FACS Analysis
  - Mass Spectrometry

- QUANTIFICATION AND STATISTICAL ANALYSIS
  - Analysis of ChIP/BrdU IP-on-chip Data
  - Average Binding/BrdU-incorporation Profiling
- DATA AND CODE AVAILABILITY

SUPPLEMENTAL INFORMATION

Supplemental Information can be found online at https://doi.org/10.1016/j.molcel.2019.08.003.

ACKNOWLEDGMENTS

We thank Cogentech for hybridization of Affymetrix chips and production of CEL data files; Hybrigenics for the Y2H screen; S.P. Bell, S. Jentsch, B. Stillman, J. Diffley, and M. Foiani for sharing reagents; D. Fachinetti and D. Menolfi for technical help with the BrdU IP-on-chip; W. Carotenuto and Mohamood Adhil for help with bioinformatics analysis; A. Cattaneo and A. Bachi for mass spectrometry analysis; F. Romeo for help with GST fusion proteins; and R. Kawasumi, M. Giannattasio, and all lab members for discussions. This work was supported by the Italian Association for Cancer Research grant IG 18976 and European Research Council consolidator grant 682190 (to D.B.) and EMBO long-term fellowship ALTF 561-2014 and an AIRC/Marie Curie Actions – COFUND iCARE fellowship (to I.P.).

AUTHOR CONTRIBUTIONS

I.P. and D.B. designed the study, analyzed the data, and wrote the paper. I.P. and F.C. conducted the experiments.

DECLARATION OF INTERESTS

The authors declare no competing interests.

Received: February 19, 2019
Revised: July 1, 2019
Accepted: August 1, 2019
Published: September 10, 2019

REFERENCES

Baldwin, M.L., Julius, J.A., Tang, X., Wang, Y., and Bachant, J. (2009). The yeast SUMO isopeptidase Smt4/Ulp2 and the polo kinase Cdc5 act in an opposing fashion to regulate sumoylation in mitosis and cohesion at centromeres. Cell Cycle *8*, 3406–3419.

Bell, S.P., and Labib, K. (2016). Chromosome Duplication in Saccharomyces cerevisiae. Genetics *203*, 1027–1067.

Bergink, S., Ammon, T., Kern, M., Schermelleh, L., Leonhardt, H., and Jentsch, S. (2013). Role of Cdc48/p97 as a SUMO-targeted segregase curbing Rad51-Rad52 interaction. Nat. Cell Biol. *15*, 526–532.

Bermejo, R., Capra, T., Gonzalez-Huici, V., Fachinetti, D., Cocito, A., Natoli, G., Katou, Y., Mori, H., Kurokawa, K., Shirahige, K., and Foiani, M. (2009). Genome-organizing factors Top2 and Hmo1 prevent chromosome fragility at sites of S phase transcription. Cell *138*, 870–884.

Blake, D., Luke, B., Kanellis, P., Jorgensen, P., Goh, T., Penfold, S., Breitkreutz, B.J., Durocher, D., Peter, M., and Tyers, M. (2006). The F-box protein Dia2 overcomes replication impedance to promote genome stability in Saccharomyces cerevisiae. Genetics *174*, 1709–1727.

Bowers, J.L., Randell, J.C.W., Chen, S., and Bell, S.P. (2004). ATP hydrolysis by ORC catalyzes reiterative Mcm2-7 assembly at a defined origin of replication. Mol. Cell *16*, 967–978.

Bylebyl, G.R., Belichenko, I., and Johnson, E.S. (2003). The SUMO isopeptidase Ulp2 prevents accumulation of SUMO chains in yeast. J. Biol. Chem. *278*, 44113–44120.

Chen, Y.C., Kenworthy, J., Gabrielse, C., Hänni, C., Zegerman, P., and Weinreich, M. (2013). DNA replication checkpoint signaling depends on a Rad53-Dbf4 N-terminal interaction in Saccharomyces cerevisiae. Genetics *194*, 389–401.

Cheng, L., Collyer, T., and Hardy, C.F. (1999). Cell cycle regulation of DNA replication initiator factor Dbf4p. Mol. Cell. Biol. *19*, 4270–4278.

Cheng, C.H., Lo, Y.H., Liang, S.S., Ti, S.C., Lin, F.M., Yeh, C.H., Huang, H.Y., and Wang, T.F. (2006). SUMO modifications control assembly of synaptonemal complex and polycomplex in meiosis of Saccharomyces cerevisiae. Genes Dev. *20*, 2067–2081.

Cox, J., and Mann, M. (2008). MaxQuant enables high peptide identification rates, individualized p.p.b.-range mass accuracies and proteome-wide protein quantification. Nat. Biotechnol. *26*, 1367–1372.

Cremona, C.A., Sarangi, P., Yang, Y., Hang, L.E., Rahman, S., and Zhao, X. (2012). Extensive DNA damage-induced sumoylation contributes to replication and repair and acts in addition to the mec1 checkpoint. Mol. Cell *45*, 422–432.

Dantuma, N.P., and Hoppe, T. (2012). Growing sphere of influence: Cdc48/p97 orchestrates ubiquitin-dependent extraction from chromatin. Trends Cell Biol. *22*, 483–491.

Dershowitz, A., and Newlon, C.S. (1993). The effect on chromosome stability of deleting replication origins. Mol. Cell. Biol. *13*, 391–398.

Eckhoff, J., and Dohmen, R.J. (2015). In vitro studies reveal a sequential mode of chain processing by the yeast SUMO (Small Ubiquitin-related Modifier)-specific protease Ulp2. J. Biol. Chem. *290*, 12268–12281.

Elmore, Z.C., Donaher, M., Matson, B.C., Murphy, H., Westerbeck, J.W., and Kerscher, O. (2011). Sumo-dependent substrate targeting of the SUMO protease Ulp1. BMC Biol. *9*, 74.

Ferreira, M.F., Santocanale, C., Drury, L.S., and Diffley, J.F.X. (2000). Dbf4p, an essential S phase-promoting factor, is targeted for degradation by the anaphase-promoting complex. Mol. Cell. Biol. *20*, 242–248.

Fiorani, S., Mimun, G., Caleca, L., Piccini, D., and Pellicioli, A. (2008). Characterization of the activation domain of the Rad53 checkpoint kinase. Cell Cycle *7*, 493–499.

Flotho, A., and Melchior, F. (2013). Sumoylation: a regulatory protein modification in health and disease. Annu. Rev. Biochem. *82*, 357–385.

Giannattasio, M., Zwicky, K., Follonier, C., Foiani, M., Lopes, M., and Branzei, D. (2014). Visualization of recombination-mediated damage bypass by template switching. Nat. Struct. Mol. Biol. *21*, 884–892.

Golebiowski, F., Matic, I., Tatham, M.H., Cole, C., Yin, Y., Nakamura, A., Cox, J., Barton, G.J., Mann, M., and Hay, R.T. (2009). System-wide changes to SUMO modifications in response to heat shock. Sci. Signal. *2*, ra24.

Hendriks, I.A., Lyon, D., Young, C., Jensen, L.J., Vertegaal, A.C., and Nielsen, M.L. (2017). Site-specific mapping of the human SUMO proteome reveals co-modification with phosphorylation. Nat. Struct. Mol. Biol. *24*, 325–336.

Hickey, C.M., Wilson, N.R., and Hochstrasser, M. (2012). Function and regulation of SUMO proteases. Nat. Rev. Mol. Cell Biol. *13*, 755–766.

Hughes, S., Elustondo, F., Di Fonzo, A., Leroux, F.G., Wong, A.C., Snijders, A.P., Matthews, S.J., and Cherepanov, P. (2012). Crystal structure of human CDC7 kinase in complex with its activator DBF4. Nat. Struct. Mol. Biol. *19*, 1101–1107.

James, P., Halladay, J., and Craig, E.A. (1996). Genomic libraries and a host strain designed for highly efficient two-hybrid selection in yeast. Genetics *144*, 1425–1436.

Janke, C., Magiera, M.M., Rathfelder, N., Taxis, C., Reber, S., Maekawa, H., Moreno-Borchart, A., Doenges, G., Schwob, E., Schiebel, E., and Knop, M. (2004). A versatile toolbox for PCR-based tagging of yeast genes: new fluorescent proteins, more markers and promoter substitution cassettes. Yeast *21*, 947–962.

Jentsch, S., and Psakhye, I. (2013). Control of nuclear activities by substrate-selective and protein-group SUMOylation. Annu. Rev. Genet. *47*, 167–186.

Kent, W.J., Sugnet, C.W., Furey, T.S., Roskin, K.M., Pringle, T.H., Zahler, A.M., and Haussler, D. (2002). The human genome browser at UCSC. Genome Res. *12*, 996–1006.

Køhler, J.B., Tammsalu, T., Jørgensen, M.M., Steen, N., Hay, R.T., and Thon, G. (2015). Targeting of SUMO substrates to a Cdc48-Ufd1-Npl4 segregase and STUbL pathway in fission yeast. Nat. Commun. *6*, 8827.

Kotecha, N., Krutzik, P.O., and Irish, J.M. (2010). Web-based analysis and publication of flow cytometry experiments. Curr. Protoc. Cytom. *Chapter 10*, Unit 10.17.

Lamoliatte, F., Caron, D., Durette, C., Mahrouche, L., Maroui, M.A., Caron-Lizotte, O., Bonneil, E., Chelbi-Alix, M.K., and Thibault, P. (2014). Large-scale analysis of lysine SUMOylation by SUMO remnant immunoaffinity profiling. Nat. Commun. *5*, 5409.

Liang, J., Singh, N., Carlson, C.R., Albuquerque, C.P., Corbett, K.D., and Zhou, H. (2017). Recruitment of a SUMO isopeptidase to rDNA stabilizes silencing complexes by opposing SUMO targeted ubiquitin ligase activity. Genes Dev. *31*, 802–815.

Livak, K.J., and Schmittgen, T.D. (2001). Analysis of relative gene expression data using real-time quantitative PCR and the 2(-Delta Delta C(T)) Method. Methods *25*, 402–408.

Mann, M. (2006). Functional and quantitative proteomics using SILAC. Nat. Rev. Mol. Cell Biol. *7*, 952–958.

Maric, M., Maculins, T., De Piccoli, G., and Labib, K. (2014). Cdc48 and a ubiquitin ligase drive disassembly of the CMG helicase at the end of DNA replication. Science *346*, 1253596.

Mark, K.G., Simonetta, M., Maiolica, A., Seller, C.A., and Toczyski, D.P. (2014). Ubiquitin ligase trapping identifies an SCF(Saf1) pathway targeting unprocessed vacuolar/lysosomal proteins. Mol. Cell *53*, 148–161.

Marmorstein, R., Carey, M., Ptashne, M., and Harrison, S.C. (1992). DNA recognition by GAL4: structure of a protein-DNA complex. Nature *356*, 408–414.

Moreno, S.P., Bailey, R., Campion, N., Herron, S., and Gambus, A. (2014). Polyubiquitylation drives replisome disassembly at the termination of DNA replication. Science *346*, 477–481.

Mullen, J.R., Das, M., and Brill, S.J. (2011). Genetic evidence that polysumoylation bypasses the need for a SUMO-targeted Ub ligase. Genetics *187*, 73–87.

Newlon, C.S., Lipchitz, L.R., Collins, I., Deshpande, A., Devenish, R.J., Green, R.P., Klein, H.L., Palzkill, T.G., Ren, R.B., Synn, S., et al. (1991). Analysis of a circular derivative of Saccharomyces cerevisiae chromosome III: a physical map and identification and location of ARS elements. Genetics *129*, 343–357.

Ohkuni, K., Takahashi, Y., Fulp, A., Lawrimore, J., Au, W.C., Pasupala, N., Levy-Myers, R., Warren, J., Strunnikov, A., Baker, R.E., et al. (2016). SUMO-Targeted Ubiquitin Ligase (STUbL) Slx5 regulates proteolysis of centromeric histone H3 variant Cse4 and prevents its mislocalization to euchromatin. Mol. Biol. Cell *27*, 1500–1510.

Poli, J., Tsaponina, O., Crabbé, L., Keszthelyi, A., Pantesco, V., Chabes, A., Lengronne, A., and Pasero, P. (2012). dNTP pools determine fork progression and origin usage under replication stress. EMBO J. *31*, 883–894.

Psakhye, I., and Jentsch, S. (2012). Protein group modification and synergy in the SUMO pathway as exemplified in DNA repair. Cell *151*, 807–820.

Psakhye, I., and Jentsch, S. (2016). Identification of substrates of protein-group SUMOylation. Methods Mol. Biol. *1475*, 219–231.

Randell, J.C., Fan, A., Chan, C., Francis, L.I., Heller, R.C., Galani, K., and Bell, S.P. (2010). Mec1 is one of multiple kinases that prime the Mcm2-7 helicase for phosphorylation by Cdc7. Mol. Cell *40*, 353–363.

Rossi, S.E., Ajazi, A., Carotenuto, W., Foiani, M., and Giannattasio, M. (2015a). Rad53-mediated regulation of Rrm3 and Pif1 DNA helicases contributes to prevention of aberrant fork transitions under replication stress. Cell Rep. *13*, 80–92.

Rossi, S.E., Carotenuto, W., and Giannattasio, M. (2015b). Genome-wide localization of Rrm3 and Pif1 DNA helicases at stalled active and inactive DNA replication forks of Saccharomyces cerevisiae. Genom. Data *7*, 162–165.

Searle, B.C. (2010). Scaffold: a bioinformatic tool for validating MS/MS-based proteomic studies. Proteomics *10*, 1265–1269.

Sherman, F. (1991). Getting started with yeast. Methods Enzymol. *194*, 3–21.

Sheu, Y.J., and Stillman, B. (2010). The Dbf4-Cdc7 kinase promotes S phase by alleviating an inhibitory activity in Mcm4. Nature *463*, 113–117.

Shin, H., Liu, T., Manrai, A.K., and Liu, X.S. (2009). CEAS: cis-regulatory element annotation system. Bioinformatics *25*, 2605–2606.

Skilton, A., Ho, J.C., Mercer, B., Outwin, E., and Watts, F.Z. (2009). SUMO chain formation is required for response to replication arrest in S. pombe. PLoS ONE *4*, e6750.

Song, J., Durrin, L.K., Wilkinson, T.A., Krontiris, T.G., and Chen, Y. (2004). Identification of a SUMO-binding motif that recognizes SUMO-modified proteins. Proc. Natl. Acad. Sci. USA *101*, 14373–14378.

Srikumar, T., Lewicki, M.C., Costanzo, M., Tkach, J.M., van Bakel, H., Tsui, K., Johnson, E.S., Brown, G.W., Andrews, B.J., Boone, C., et al. (2013). Global analysis of SUMO chain function reveals multiple roles in chromatin regulation. J. Cell Biol. *201*, 145–163.

Sriramachandran, A.M., and Dohmen, R.J. (2014). SUMO-targeted ubiquitin ligases. Biochim. Biophys. Acta *1843*, 75–85.

Uzunova, K., Göttsche, K., Miteva, M., Weisshaar, S.R., Glanemann, C., Schnellhardt, M., Niessen, M., Scheel, H., Hofmann, K., Johnson, E.S., et al. (2007). Ubiquitin-dependent proteolytic control of SUMO conjugates. J. Biol. Chem. *282*, 34167–34175.

Vertegaal, A.C. (2010). SUMO chains: polymeric signals. Biochem. Soc. Trans. *38*, 46–49.

Wang, Z., Jones, G.M., and Prelich, G. (2006). Genetic analysis connects SLX5 and SLX8 to the SUMO pathway in Saccharomyces cerevisiae. Genetics *172*, 1499–1509.

Wei, L., and Zhao, X. (2016). A new MCM modification cycle regulates DNA replication initiation. Nat. Struct. Mol. Biol. *23*, 209–216.

Xie, Y., Rubenstein, E.M., Matt, T., and Hochstrasser, M. (2010). SUMO-independent in vivo activity of a SUMO-targeted ubiquitin ligase toward a short-lived transcription factor. Genes Dev. *24*, 893–903.

Zegerman, P., and Diffley, J.F. (2007). Phosphorylation of Sld2 and Sld3 by cyclin-dependent kinases promotes DNA replication in budding yeast. Nature *445*, 281–285.

STAR★METHODS

KEY RESOURCES TABLE

REAGENT or RESOURCE	SOURCE	IDENTIFIER
Antibodies		
Mouse monoclonal anti-FLAG antibody (clone M2) (Dilution for western blot 1:2000)	Sigma-Aldrich	Cat# F1804; RRID: AB_262044
Mouse monoclonal anti-Viral V5-TAG antibody (clone SV5-Pk1) (Dilution for western blot 1:5000)	Bio-Rad / AbD Serotec	Cat# MCA1360; RRID: AB_322378
Mouse monoclonal anti-Pgk1 antibody (clone 22C5D8) (Dilution for western blot 1:2000)	Thermo Fisher Scientific	Cat# 459250; RRID: AB_2532235
Mouse monoclonal anti c-MYC antibody (clone 9E10) (Dilution for western blot 1:2000)	In house	N/A
Mouse monoclonal anti-Rad53 antibody (clone EL7) (Dilution for western blot 1:5)	In house (Fiorani et al., 2008)	N/A
Rabbit polyclonal anti-GST antibody (Dilution for western blot 1:3000)	In house	N/A
Mouse monoclonal anti-HA (F-7) antibody (Dilution for western blot 1:2000)	Santa Cruz Biotechnology	Cat# sc-7392; RRID: AB_627809
Mouse monoclonal anti-Bromodeoxyuridine antibody (clone 2B1)	MBL International	Cat# MI-11-3; RRID: AB_590678
Rabbit polyclonal anti-Ubiquitin antibody (Dilution for western blot 1:2000)	Abcam	Cat# ab19247; RRID: AB_444805
Rabbit polyclonal anti-Clb2 (y-180) antibody (Dilution for western blot 1:2000)	Santa Cruz Biotechnology	Cat# sc-9071; RRID: AB_667962
Rabbit polyclonal anti-Smt3 (y-84) antibody (Dilution for western blot 1:2000)	Santa Cruz Biotechnology	Cat# sc-28649; RRID: AB_661135
Rabbit polyclonal anti-SUMO2/3 antibody (Dilution for western blot 1:2000)	Abcam	Cat# ab3742; RRID: AB_304041
Rabbit polyclonal anti-Mcm2-7 (UM185) antibody (Dilution for western blot 1:5000)	Gift from Stephen P. Bell (Bowers et al., 2004)	N/A
Rabbit polyclonal anti-Mcm4-phospho-S82-D83 antibody (Dilution for western blot 1:400)	Gift from Stephen P. Bell (Randell et al., 2010)	N/A
Anti-HA affinity matrix; (clone 3F10) rat monoclonal antibody	Roche	Cat# 11815016001; RRID: AB_390914
Anti-rabbit IgG, HRP-linked antibody (Dilution for western blot 1:5000)	Cell Signaling Technology	Cat# 7074; RRID: AB_2099233
Anti-mouse IgG, HRP-linked antibody (Dilution for western blot 1:5000)	Cell Signaling Technology	Cat# 7076; RRID: AB_330924
Normal mouse IgG	Santa Cruz Biotechnology	Cat# sc-2025; RRID: AB_737182
Chemicals, Peptides, and Recombinant Proteins		
alpha-factor mating pheromone (WHWLQLKPGQPMY)	GenScript; RRID: SCR_002891	Cat# 59401-28-4
Recombinant human poly-SUMO3 wild-type K-11-linked chains (2-8)	Boston Biochem	Cat# ULC-310
Recombinant budding yeast N-terminally His-tagged wild-type SUMO (^{His}SUMO)	In house	N/A
Recombinant glutathione S-transferase (GST) and GST-Ulp2 fusion proteins (amino acids 1-400, wild-type or with mutated SUMO-interacting motifs)	In house	N/A
Nocodazole	Sigma-Aldrich	Cat# M1404
Imidazole	Sigma-Aldrich	Cat# I2399

(*Continued on next page*)

Continued

REAGENT or RESOURCE	SOURCE	IDENTIFIER
Hydroxyurea	Sigma-Aldrich	Cat# H8627
Bromodeoxyuridine	Sigma-Aldrich	Cat# B9285
Ni-NTA agarose	QIAGEN	Cat# 30210
Recombinant protein G – Sepharose 4B	Thermo Fisher Scientific	Cat# 101243
Glutathione Sepharose 4B	GE Healthcare	Cat# 17-0756-01
Dynabeads protein A	Thermo Fisher Scientific	Cat# 10002D
cOmplete, EDTA-free protease inhibitor cocktail tablets	Roche	Cat# 4693132001
N-Ethylmaleimide	Sigma-Aldrich	Cat# E3876
Phenylmethanesulfonyl fluoride	Sigma-Aldrich	Cat# P7626
Iodoacetamide	Sigma-Aldrich	Cat# I1149
Phosphatase inhibitor cocktail 2	Sigma-Aldrich	Cat# P5726
Phosphatase inhibitor cocktail 3	Sigma-Aldrich	Cat# P0044
Zymolyase 100T (Arthrobacter luteus)	Seikagaku Corporation	Cat# 120493
Lambda protein phosphatase	New England Biolabs	Cat# P0753S
Ribonuclease A from bovine pancreas	Sigma-Aldrich	Cat# R5503
Proteinase K, recombinant, PCR Grade	Roche	Cat# 03115801001
4,5′,8-Trimethylpsoralen	Sigma-Aldrich	Cat# T6137
Agarose D1-LE	Fisher Molecular Biology	Cat# AS-101
Critical Commercial Assays		
GenomePlex complete whole genome amplification (WGA) kit	Sigma-Aldrich	Cat# WGA2
GenomePlex WGA reamplification kit	Sigma-Aldrich	Cat# WGA3
QuantiFast SYBR Green PCR kit	QIAGEN	Cat# 204054
Genomic-tip 100/G	QIAGEN	Cat# 10243
QIAquick PCR purification kit	QIAGEN	Cat# 28106
ProbeQuant G-50 micro columns	GE Healthcare	Cat# 28903408
Prime-a-Gene labeling system	Promega	Cat# U1100
Invitrogen Bolt 4-12% Bis-Tris Plus Gels, 15-well	Thermo Fisher Scientific	Cat# NW04125BOX
L-Arginine:HCl (U-13C6, 99%; U-15N4, 99%)	Cambridge Isotope Laboratories	CAS# 1119-34-2<br>CNLM-539-H-0.25
L-Lysine:2HCl (U-13C6, 99%; U-15N2, 99%)	Cambridge Isotope Laboratories	CAS# 657-26-1<br>CNLM-291-H-0.25
Deposited Data		
Raw and analyzed ChIP-on-chip and BrdU IP-on-chip data	This paper	GEO: GSE113835
Experimental Models: Organisms/Strains		
All yeast Saccharomyces cerevisiae strains used in this work, except those used for yeast two-hybrid (Y2H) studies, are W303 background derivatives with the wild type *RAD5* locus. They are listed in Table S2.	This paper	N/A
Y2HGold yeast strain	Takara	Cat# 630498
Oligonucleotides		
Primer ARS305F for qPCR: CTCCGTTTTTAGCC CCCGTG	This paper	N/A
Primer ARS305R for qPCR: GATTGAGGCCACAGCAAGACCG	This paper	N/A

(*Continued on next page*)

Continued		
REAGENT or RESOURCE	SOURCE	IDENTIFIER
Recombinant DNA		
A6C-110	Newlon et al., 1991	N/A
pGAD-C1, pGBD-C1	James et al., 1996	N/A
pGEX-6P-2	GE Healthcare	Cat# 28-9546-50
Software and Algorithms		
Affymetrix Tiling Analysis Software	Thermo Fisher Scientific	https://www.thermofisher.com/us/en/home/life-science/microarray-analysis/microarray-analysis-instruments-software-services/microarray-analysis-software/tiling-array-tools.html
UCSC Genome Browser	Kent et al., 2002	https://genome.ucsc.edu/
CEAS (Cis-regulatory Element Annotation System) package sitepro script	Shin et al., 2009	http://liulab.dfci.harvard.edu/CEAS/download.html
Cytobank	Kotecha et al., 2010	https://www.cytobank.org/
MaxQuant (version 1.5.2.8)	Cox and Mann, 2008	https://www.biochem.mpg.de/5111795/maxquant
Scaffold	Searle, 2010	http://www.proteomesoftware.com/products/scaffold/
Other		
ULTImate yeast two-hybrid (Y2H) screen	Hybrigenics Services	https://www.hybrigenics-services.com/

LEAD CONTACT AND MATERIALS AVAILABILITY

Further information and requests for resources and reagents should be directed to and will be fulfilled by the Lead Contact, Dana Branzei (dana.branzei@ifom.eu).

EXPERIMENTAL MODEL AND SUBJECT DETAILS

Yeast Strains

Chromosomally tagged *Saccharomyces cerevisiae* strains and mutants were constructed by a PCR-based strategy, by genetic crosses and standard techniques (Janke et al., 2004). Standard cloning and site-directed mutagenesis techniques were used. Strains and all genetic manipulations were verified by polymerase chain reaction (PCR), sequencing and phenotype. Maps and primer DNA sequences are available upon request. All yeast strains used in this work except those used for the yeast two-hybrid (Y2H) studies are isogenic to W303 background and are listed in the Key Resources Table.

METHOD DETAILS

Yeast Techniques

Yeast cultures were inoculated from overnight cultures, grown using standard growth conditions and media (Sherman, 1991). All cultures were grown in YPD-media containing glucose (2%) as carbon source at 28°C unless otherwise indicated. For cell cycle synchronization, logarithmic cells grown at 28°C were arrested in G1 using 3-5 μg/ml of alpha-factor for 2-3 hours. G2 arrest was performed with 20 μg/ml of nocodazole for 2-3 hours. G1/G2-arrest was verified microscopically and by FACS analysis. Hydroxyurea (HU) was used at the concentration of 200 mM, bromodeoxyuridine (BrdU) at 200 μg/ml. For drug sensitivity assays, cells from overnight cultures were counted and diluted before being spotted on YPD plates containing the indicated concentrations of HU and incubated at 28°C for 2-3 days. The ULTImate Y2H screen using full-length Ulp2 (N-GAL4-ULP2-C fusion) as bait was performed by Hybrigenics Services. For further Y2H analysis *CDC7*, *DBF4* and different truncations/mutants of *ULP2* were cloned into pGAD-C1 or pGBD-C1 vectors and cotransformed into Y2HGold yeast strain. Standard cloning and site-directed mutagenesis techniques were used. Maps and primer DNA sequences are available upon request.

TCA Protein Precipitation

To preserve the post-translational modifications, yeast cells were lysed under denaturing conditions. For preparation of denatured protein extracts, yeast cultures grown to an OD_{600} = 0.7-1 were pelleted by centrifugation (4000 rpm, 4 min, 4°C) and immediately frozen in liquid nitrogen. After thawing on ice, the pellets were lysed by addition of denaturing lysis buffer

(1.85 M NaOH, 7.5% β-mercaptoethanol) for 15min on ice. For the cell pellet of an OD_{600} = 1 typically 150 μL of lysis buffer was used. To precipitate the proteins, the lysate was subsequently mixed with an equal volume (150 μL in case of OD_{600} = 1) of 55% (w/v) trichloroacetic acid (TCA) and further incubated on ice for 15 min. The precipitated material was recovered by two sequential centrifugation steps (13000 rpm, 4°C, 15 min). Pelleted denatured proteins were then either directly resuspended in HU sample buffer (8 M urea, 5% SDS, 1 mM EDTA, 1.5% DTT, 1% bromophenol blue; 50 μL per OD_{600} = 1), boiled for 10 min and stored at −20°C, or used for downstream processing, e.g., Ni-NTA pull-downs of His-tagged SUMO conjugates.

Ni-NTA Pull-down of ^{His}SUMO Conjugates

For isolation of *in vivo* SUMOylated substrates from yeast cells expressing N-terminally His-tagged Smt3 (^{His}SUMO), denatured protein extracts were prepared and Ni-NTA chromatography was carried out as described previously (Psakhye and Jentsch, 2012, 2016). In general, 200 OD_{600} = 1 of logarithmically growing cells were harvested by centrifugation (4000 rpm, 4 min, 4°C), washed with pre-chilled water, transferred to 50 mL falcon tube and lysed with 6 mL of 1.85 M NaOH / 7.5% β-mercaptoethanol for 15 min on ice. The proteins were precipitated by adding 6 mL of 55% TCA and another 15 min incubation on ice (TCA-precipitation, described above). Next, the precipitate was pelleted by centrifugation (3500 rpm, 15 min, 4°C), washed twice with water and finally resuspended in buffer A (6 M guanidine hydrochloride, 100 mM NaH_2PO_4, 10 mM Tris-HCl, pH 8.0, 20 mM imidazole) containing 0.05% Tween-20. After incubation for 1 hour on a roller at room temperature with subsequent removal of insoluble aggregates by centrifugation (23000 g, 20 min, 4°C), the protein solution was incubated overnight at 4°C with 50 μL of Ni-NTA agarose beads in the presence of 20 mM imidazole. After incubation, the beads were washed three times with buffer A containing 0.05% Tween-20 and five times with buffer B (8 M urea, 100 mM NaH_2PO_4, 10 mM Tris-HCl, pH 6.3) with 0.05% Tween-20. ^{His}SUMO conjugates bound to the beads were finally eluted by incubation with 50 μL of HU sample buffer for 10 min at 65°C. Proteins were resolved on precast Bolt 4%–12% Bis-Tris Plus gradient gels, and analyzed by standard western blotting techniques using antibodies listed in the Key Resources Table.

Immunoprecipitation and Phosphatase Treatment

For the immunoprecipitation (IP), IPs followed by lambda protein phosphatase treatment, and binding studies involving co-IP, native yeast extracts were prepared by cell disruption using grinding in liquid nitrogen. To avoid protein degradation and loss of PTMs, lysis buffer (150 mM NaCl, 10% glycerol, 1% NP-40, 50 mM Tris HCl, pH 8.0) was supplemented with inhibitors: EDTA-free complete cocktail, 20mM N-ethylmaleimide, 1 mM phenylmethanesulfonyl fluoride (PMSF), 25 mM iodoacetamide, and phosphatase inhibitor cocktails 2 and 3 (Sigma-Aldrich). For IPs, anti-PK, anti-FLAG, or anti-MYC antibodies, together with recombinant protein G Sepharose 4B beads, or anti-HA affinity matrix alone were used. IPs were performed overnight with head-over-tail rotation at 4°C and were followed by stringent washing steps to remove non-specific background binding to the beads. For protein dephosphorylation, lambda protein phosphatase was used to treat IP samples following Sepharose bead washing according to manufacturer's instructions, and was either supplemented with phosphatase inhibitor cocktails 2 and 3 (Sigma-Aldrich) to inactivate the phosphatase or not.

GST *in vitro* pull-down assays

The N terminus of Ulp2 (aa 1-400) either wild-type or with different SIMs mutated (Ulp2-V60A,L61A,L200A,L201A, L206A,I207A,I208A, termed Ulp2-sim1,2,3; Ulp2-V60A,L61A,L200A,L201A,L206A,I207A,I208A,I229A,L230A,I276A,L277A, termed Ulp2-sim1,2,3,4,5) was cloned into pGEX-6P-2 (GE Healthcare) vector, and Rosetta(DE3) pLysS competent *E. coli* cells (Novagen) were used for GST-fusion protein expression. Following overnight protein induction with 0.25 mM IPTG at 16°C in 500 mL cell cultures, cells were pelleted, resuspended in 30 mL lysis buffer (1X PBS, 500 mM NaCl, 1% Triton X-100, lysozyme, Calbiochem EDTA-free protease inhibitor cocktail set III) and sonicated on ice. The crude lysate was clarified by centrifugation at 15000 rpm for 15 min at 4°C, and the supernatant was mixed with 0.2 mL of glutathione Sepharose 4B beads (GE Healthcare) pre-equilibrated with lysis buffer. Following overnight incubation at 4°C, five washes with lysis buffer were performed and the beads with bound GST-fusion proteins were used for subsequent *in vitro* pull-down assays with recombinant yeast N-terminally His-tagged Smt3 and human poly-SUMO3 wild-type chains (2-8 moieties) from BostonBiochem. The amounts of GST-fusion proteins bound to the beads were estimated by comparison to the BSA samples of known concentrations resolved by SDS-PAGE and Coomassie Blue staining. To study the interaction between GST-$Ulp2_{1-400}$ fusions and SUMO, either purified recombinant His-tagged yeast Smt3 (2.5 μg) or recombinant human poly-SUMO3 wild-type (2-8 moieties) chains (2.5 μg) were incubated either with GST-$Ulp2_{1-400}$ (2.5 μg) and its SIM mutant variants, or GST (2.5 μg) alone bound to glutathione Sepharose 4B beads in 0.7 mL of binding buffer (1X PBS, 150 mM NaCl, 1% Triton X-100, Calbiochem EDTA-free protease inhibitor cocktail set III) overnight at 4°C, with gentle mixing. After the incubation, the beads were washed 5 times with 1 mL of the binding buffer and bound proteins were eluted with 50 μL of HU sample buffer. Samples were then analyzed by SDS-PAGE followed by western blotting and probing with anti-Smt3, anti-SUMO2/3 and anti-GST antibodies, and subsequent staining of the membrane with Ponceau S.

ChIP, ChIP-on-chip, BrdU IP-on-chip

Chromatin immunoprecipitation (ChIP) was carried out as previously described (Bermejo et al., 2009). Briefly, cells were collected at the indicated experimental conditions and crosslinked with 1% formaldehyde for 15-30 min. Cells were washed twice with ice-cold

1X TBS, suspended in lysis buffer supplemented with 1 mM PMSF and 1X EDTA-free complete cocktail, and lysed using FastPrep-24 (MP Biomedicals). Chromatin was sheared to a size of 300-500 bp by sonication. IP reactions, with anti-FLAG or anti-PK antibodies and Dynabeads protein A, were allowed to proceed overnight at 4°C. After washing and eluting the ChIP fractions from beads, crosslinks were reversed at 65°C overnight for both SUP and IP. After proteinase K treatment, DNA was extracted twice by phenol/chlorophorm/isoamyl alcohol (25:24:1, v/v). Following precipitation with ethanol and Ribonuclease A (RNase A) treatment, DNA was purified using QIAquick PCR purification kit. For ChIP-on-chip, DNA was then amplified using GenomePlex complete whole genome amplification (WGA) kits WGA2 and WGA3 following manufacturer's instructions. 4 μg of DNA from SUP and IP samples were hybridized to GeneChip *S. cerevisiae* Tiling 1.0R Array (Affymetrix). For the BrdU IP experiments, the genomic DNA extraction was performed using the Genomic-tip 100/G columns, followed by sonication to shear the DNA to a size of 300-500 bp. The IP of the BrdU-containing DNA was performed using the anti-BrdU antibody.

ChIP-qPCR

ChIP-qPCR was performed using QuantiFast SYBR Green PCR kit according to the manufacturer's instructions and each reaction was performed in triplicates using a Roche LightCycler 480 system. The results were analyzed with absolute quantification/2nd derivative maximum and the 2(-ΔC(t)) method as previously described (Livak and Schmittgen, 2001). Error bars represent standard deviations.

2D Gel Electrophoresis

Cells were synchronized in G1 phase with alpha-factor at 28°C and released in media containing HU 0.2 M. Samples were collected at the indicated time points and incubated with sodium azide 1% for 30 min on ice. *In vivo* psoralen crosslinking and DNA extraction with CTAB were performed as in Giannattasio et al. (2014). Briefly, cells were washed, resuspended in 5ml of cold water in small Petri dishes and kept on ice. 300 μL of 4,5′,8-trimethylpsoralen solution (0.2 mg/ml in EtOH 100%) was added prior to extensive resuspension by pipetting, followed by 5 min of incubation in the dark and then 10 min of UV irradiation at 365 nm (Stratagene UV Stratalinker 2400). The procedure was repeated 3 times to ensure extensive crosslinking. Cells were then harvested by centrifugation, washed in cold water, and incubated in spheroplasting buffer (1M sorbitol, 100 mM EDTA, 0.1% β-mercaptoethanol, and 50 U zymolyase/ml) for 1.5 h at 30°C. In all, 2 mL water, 200 μL RNase A (10 mg/ml), and 2.5 mL Solution I (2% w/v cetyltrimethylammonium bromide (CTAB), 1.4 M NaCl, 25 mM EDTA, 100 mM Tris–HCl, pH 7.6) were sequentially added to the spheroplast pellets and samples were incubated for 30 min at 50°C. 200 mL Proteinase K (20 mg/ml) was then added and the incubation was prolonged at 50°C for 90 min, and at 30°C overnight. The sample was then centrifuged at 4000 rpm for 10 min: the cellular debris pellet was kept for further extraction, while the supernatant was extracted with 2.5 mL chloroform/isoamylalcohol (24:1) and the DNA in the upper phase was precipitated by addition of 2 volumes of Solution II (1% w/v CTAB, 10 mM EDTA, 50 mM Tris–HCl, pH 7.6) and centrifugation at 8500 rpm for 10 min. The pellet was resuspended in 2 mL Solution III (1.4 M NaCl, 1 mM EDTA, 10 mM Tris–HCl, pH 7.6). Residual DNA in the cellular debris pellet was also extracted by resuspension in 2ml Solution III and incubation at 50°C for 30 min, followed by extraction with 1 mL chloroform/isoamylalcohol (24:1). The upper phase was pooled together with the main DNA prep. Total DNA was then precipitated with 1 volume of isopropanol, washed with 70% ethanol, air-dried, and finally resuspended in 1x TE. Alternatively, DNA can be extracted following the QIAGEN protocol for the purification of yeast genomic DNA using the Genomic-tip 100/G columns. Subsequently, 10 μg of DNA were digested with the indicated restriction endonucleases, precipitated with potassium acetate and isopropanol, and resuspended in 10mM Tris-HCl, pH 8.0. Digested genomic DNA was run on the Thermo Scientific Owl A2 large gel system (gel tray 27x20 cm) filled with 2.5 l of 1x TBE. The first dimension gel (500 ml; 0.35% w/v Agarose D1-LE) was prepared with 1x TBE and run at 50 V for 24 hours at room temperature. The second dimension gel (500 ml; 0.9% w/v Agarose D1-LE) prepared with 1x TBE was run in the same electrophoresis chamber at 150 V for 12 hours at 4°C with current limited to 150 mA. DNA molecules separated on the second dimension gels were transferred onto nylon filters via Southern blotting following standard procedures. Signals were detected using a probe against *ARS305* (BamHI-NcoI 3.0 kb fragment that spans ARS305 and was purified from plasmid A6C-110) radiolabelled according to the protocol of the Prime-A-Gene labeling system and purified with ProbeQuant G-50 micro columns.

FACS Analysis

For flow cytometry analysis, approximately 7x10^{6} cells for each time-point were collected, washed in sterile water, and permeabilized in 70% ethanol at 4°C overnight. Cells were suspended in 10 mM Tris pH 7,5 buffer, and RNA together with proteins were removed by RNase A (0,4 mg/ml final concentration) and proteinase K (1 mg/ml) treatment. Subsequently, cells were stained with PI (propidium iodide 50 μg/ml). Cell cycle profiles were obtained following a brief sonication using a Becton Dickinson FACSCalibur system. Acquired data was analyzed with Cytobank.

Mass Spectrometry

For the detection of degradation-prone SUMO conjugates decreased in abundance in *ulp2Δ cim3-1* mutant cells specifically in a SUMO-chain-dependent manner (Figure 7B), SILAC-based mass spectrometry protocol (Mann, 2006) was used. Yeast *ulp2Δ cim3-1* mutant cells deficient in biosynthesis of lysine and arginine (*lys1Δ* and *arg4Δ*) expressing either wild-type His-tagged SUMO (^{His}SUMO) or its lysine-less variant (*KRall*) that cannot form lysine-linked polySUMO chains were grown for at least ten

divisions in synthetic complete media supplemented either with unlabeled (Lys0 and Arg0; light) or heavy isotope-labeled amino acids (Lys8 and Arg10; heavy) from Cambridge Isotope Laboratories. Exponentially dividing *^{His}SUMO ulp2Δ cim3-1* cells grown in heavy media were harvested, combined with equal amount of *KRall ulp2Δ cim3-1* cells grown in light media, and SUMO conjugates were isolated by using denaturing Ni-NTA pull-down. Proteins isolated following Slx5/8 ubiquitin ligase substrate trapping (Mark et al., 2014) and denaturing Ni-NTA pull-downs of ^{His}SUMO conjugates were separated on 4%–12% Bis-Tris gel. The whole lane was excised in slices and proteins were digested with trypsin. Extracted peptides were analyzed by LC-MS/MS using the Q Exactive HF mass spectrometer and identified by either using MaxQuant (Cox and Mann, 2008) or Scaffold (Searle, 2010) software. Proteins identified using Scaffold with stringent criteria (the minimum protein probability – 99%; the minimum number of unique peptides – 2; the minimum peptide probability – 95%) are listed in Table S1, related to Figures 7A and S7E.

QUANTIFICATION AND STATISTICAL ANALYSIS

Analysis of ChIP/BrdU IP-on-chip Data

CEL files obtained by scanning of the hybridized Affymetrix chips were analyzed using a modified version of the Tiling Array Suite (TAS) software from Affymetrix as previously described (Bermejo et al., 2009). Briefly, the software performs a linear scale normalization of input CEL files (IP and SUP) intensity so that the median value is equal to a selected target intensity of 500. Signals and the p value changes obtained from TAS per each probe position are subsequently used by the software to detect clusters of enriched signals as ranges within the chromosomes. Conditions for clusters detection in whole range (at least 600 bps), except for segments within the range shorter than 600 bps, were: log2 signal (IP/SUP binding ratio) positive and change in p value (evaluated using Wilcoxon signed-rank test) less than 0.2. The peak of each cluster has been defined as the genomic position within the cluster with the highest estimated signal. All the clusters identified for all datasets produced in the work are available upon request as BED tracks suitable for visualization on the UCSC genome browser. Evaluation of the significance of protein binding/BrdU-incorporation cluster distributions within the different genomic areas (e.g., origins of replication, ARSs) and the significance of the overlap between clusters was performed by confrontation to the model of the null hypothesis distribution generated by a Monte Carlo-like simulation as previously described (Bermejo et al., 2009). Briefly, for each pair of datasets (protein binding/BrdU-incorporation clusters and a given genomic area, e.g., early origins of replication) 1000 randomizations of the positions of the genomic ranges were produced, while maintaining the following conditions at each randomization. First, the number and sizes of all the genomic areas covered within each chromosome remain the same. Second, the genomic areas are positioned in a safely random manner on the chromosome, ensuring that they do not overlap with each other. For each randomized set, the number of peaks falling within the ranges was counted and taken as a random score of the model. The distribution of random scores was validated to be approximately normal and then the average and standard deviation for the random model was taken as null hypothesis. The increase or decrease ratio for the scores of the actual positions of clusters with respect to the expected value for the null hypothesis (the average score of random attempts) was then calculated, and the p value for the drift was estimated as the cumulative distribution function of the standard normal distribution. The significance of overlap between the protein binding/BrdU-incorporation clusters (e.g., Ulp2 clusters versus BrdU-incorporation clusters) was performed following the same logic used to validate the distribution in the different genomic areas with one difference. The "score" for both the randomized positions and the actual data was calculated as the total number of overlapping bases among the whole clusters. Using the number of overlapping bases introduces a linear dependency of the obtained score from the average size of the clusters, which happens in both the randomized sets and the actual one and thus cancelled for the purpose of the simulation. Furthermore, in this case, the randomization is performed twice: once for each set, and results are evaluated independently in order to assess if there is any bias introduced by the structure of the two covered areas (sizes and spacing between the covered ranges).

Average Binding/BrdU-incorporation Profiling

Average profiling of the chromatin binding and BrdU incorporation signals within specific genomic loci was obtained using the sitepro script of CEAS (Cis-Regulatory Element Annotation System) package (Shin et al., 2009). Briefly, log2 signal (IP/SUP binding ratio) BED files obtained from the chromatin binding/BrdU incorporation analyses were converted to WIG files and used to draw the average signals around specific genomic loci, e.g., 141 active ARSs (origins of replication) as in Rossi et al. (2015b), setting 50 bps as the profiling resolution and varying sizes (1.5-22 kbps) of flanking regions from the center of each specific genomic loci. For the calculation of average chromatin binding/BrdU incorporation signals, negative values were either taken unchanged or set to zero.

DATA AND CODE AVAILABILITY

The accession number for the microarray data reported in this paper is Gene Expression Omnibus (GEO): GSE113835.

Molecular Cell, Volume *76*

Supplemental Information

SUMO-Chain-Regulated Proteasomal Degradation Timing Exemplified in DNA Replication Initiation

Ivan Psakhye, Federica Castellucci, and Dana Branzei

Figure S1

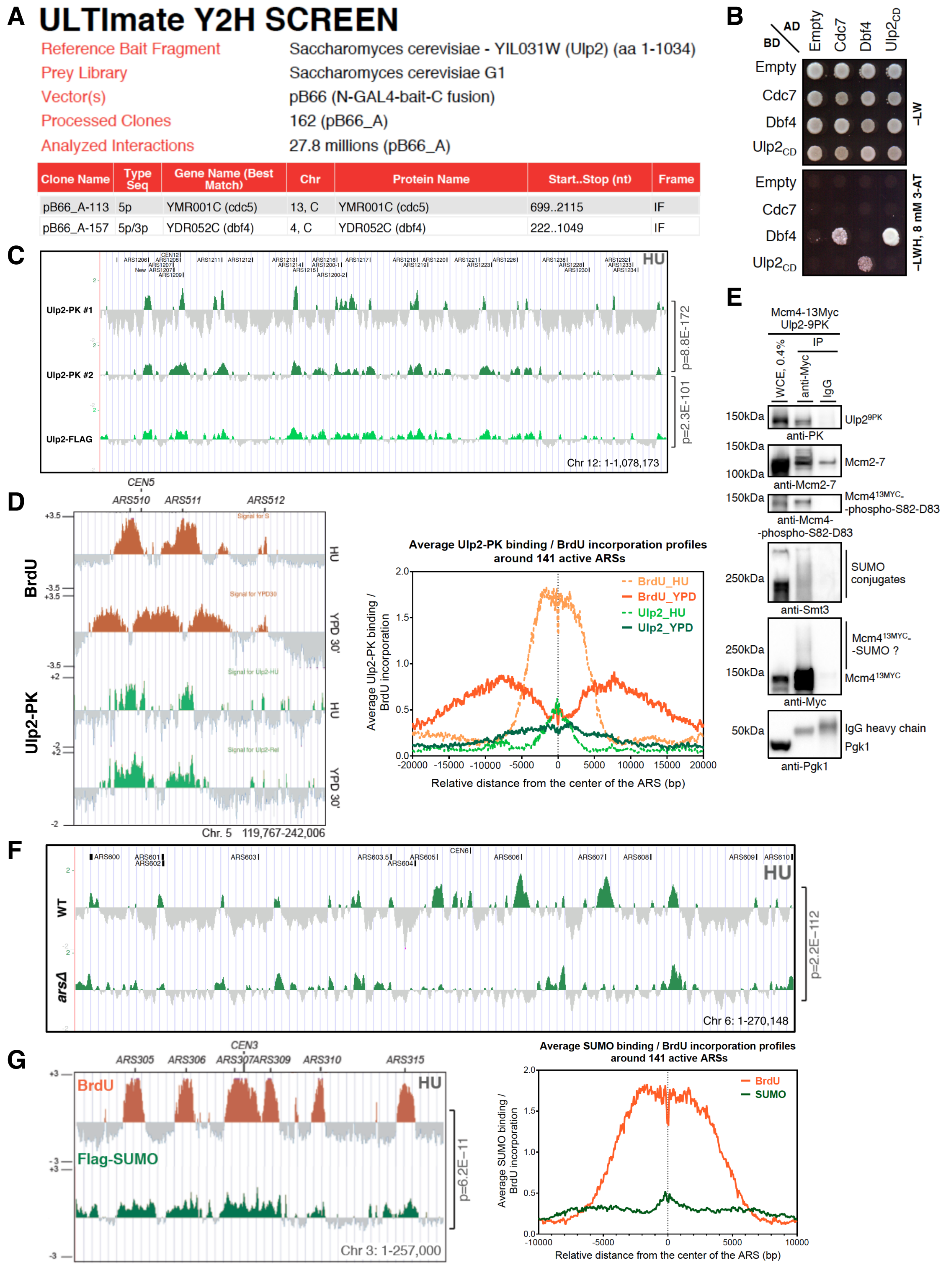

Figure S1. SUMO-deconjugating Enzyme Ulp2 Interacts with DDK and Is Recruited to Replication Origins, Related to Figure 1.

(A) The parameters of the ULTImate Y2H screen performed by Hybrigenics using full-length Ulp2 as bait. The fragments of the Polo-like kinase Cdc5 and the regulatory subunit of DDK, Dbf4, identified as interactors of Ulp2.

(B) Dbf4 interacts with Ulp2 (catalytically dead ulp2-C624S, $Ulp2_{CD}$, has stronger interaction with its substrates compared to the WT protein) in the Y2H system. 8mM 3-amino-triazole (3-AT) added to reduce auto-activation of the *HIS3* reporter gene.

(C) Overlapping ChIP-on-chip profiles of Ulp2-PK and Ulp2-FLAG from cells released into the S phase in the presence of 0.2 M HU for 90 min after G1 arrest. Chromosome 12 with annotated ARS regions and centromere is shown as an example. The p values of the genome-wide overlap between the Ulp2-PK and Ulp2-FLAG clusters, as well as between the Ulp2-PK clusters in two independent experiments are indicated.

(D) BrdU IP-on-chip profiles (orange) and ChIP-on-chip profiles of Ulp2-PK (green) from cells released into the S phase in the presence of 0.2 M HU and BrdU for 90 min after G1 arrest, and from cells subsequently released into YPD plus BrdU for 30 min following HU washout. Average Ulp2-PK binding and BrdU incorporation profiles in a window of 40 kbps centered at each of the 141 active ARSs are shown (right).

(E) Ulp2 interacts with Mcm4 subunit of the MCM helicase in Co-IP studies. Whole cell extracts (WCE) were prepared from cells expressing C-terminally 9PK-tagged Ulp2 and 13MYC-tagged Mcm4. DDK-phosphorylated Mcm4 species, other MCM subunits and $Ulp2^{9PK}$ CoIP, when $Mcm4^{13MYC}$ is immunoprecipitated with anti-Myc antibody, but not with mouse IgG.

(F) ChIP-on-chip profiles of Ulp2-PK from WT cells and cells with ARSs 604-607 being mutated (*ars*Δ**)**. Entire chromosome 6 with annotated ARS regions and centromere is shown. The p value is related to the genome-wide overlap between the Ulp2-PK clusters in the two indicated strains.

(G) SUMO conjugates are enriched at the replication origins. BrdU IP-on-chip profile (orange) and ChIP-on-chip profile of Flag-SUMO (dark green) from cells released into the S phase in the presence of 0.2 M HU and BrdU, as in (D).

Figure S2

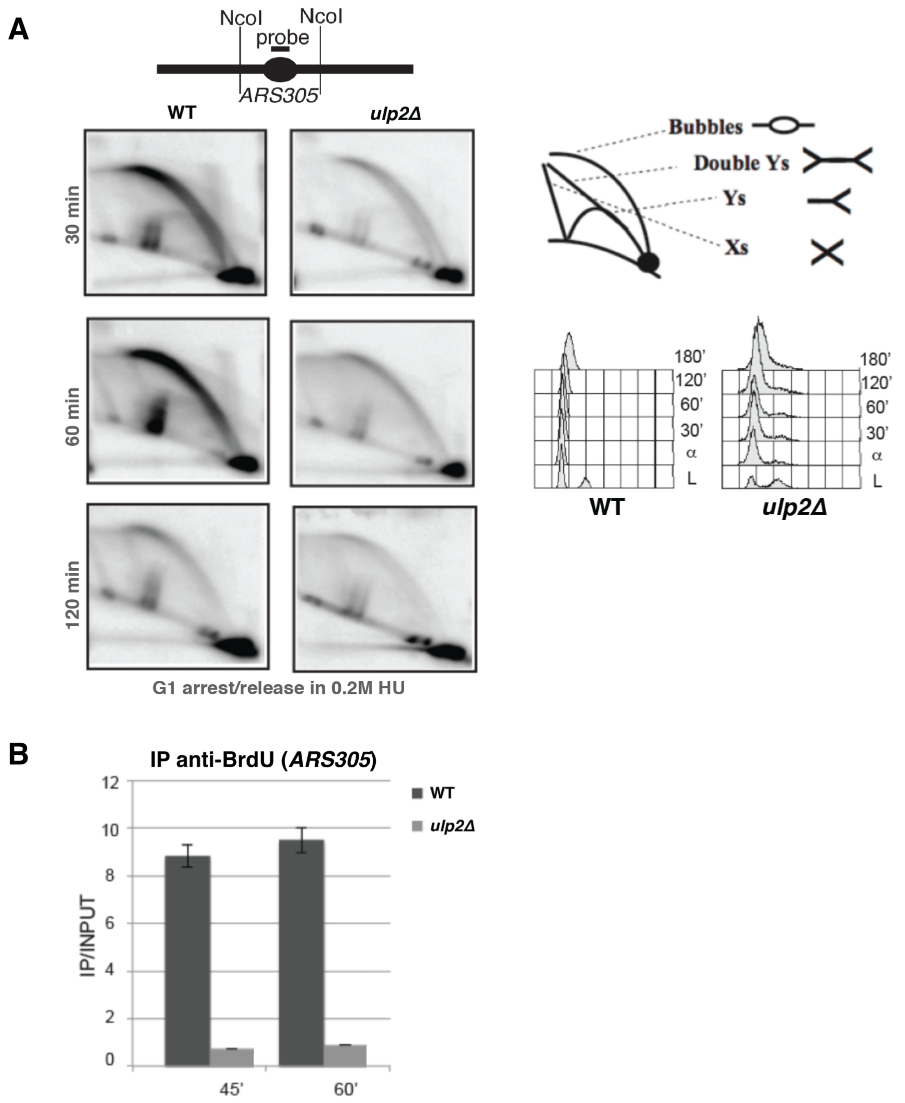

Figure S2. Accumulation of Replication Intermediates and BrdU Incorporation Are Decreased in *ulp2*Δ Cells, Related to Figure 2.

(A) The abundance of replication intermediates is decreased in cells lacking Ulp2. WT and *ulp2*Δ cells were released from G1 arrest into the S phase in the presence of 0.2 M HU for the indicated time (min), as judged from the FACS profiles (bottom right), and replication intermediates subjected to 2D gel analysis. Schematic representations of the 2D gel fragment analyzed (top) and of the type of intermediates that may be revealed by 2D gel (top right).

(B) BrdU incorporation at early replication origins is decreased in *ulp2*Δ cells compared to WT as monitored by BrdU IP-qPCR at *ARS305* in cells synchronized in G1 phase and released into the S phase in the presence of 0.2 M HU and BrdU for the indicated time (min). Error bars represent standard deviations.

Figure S3

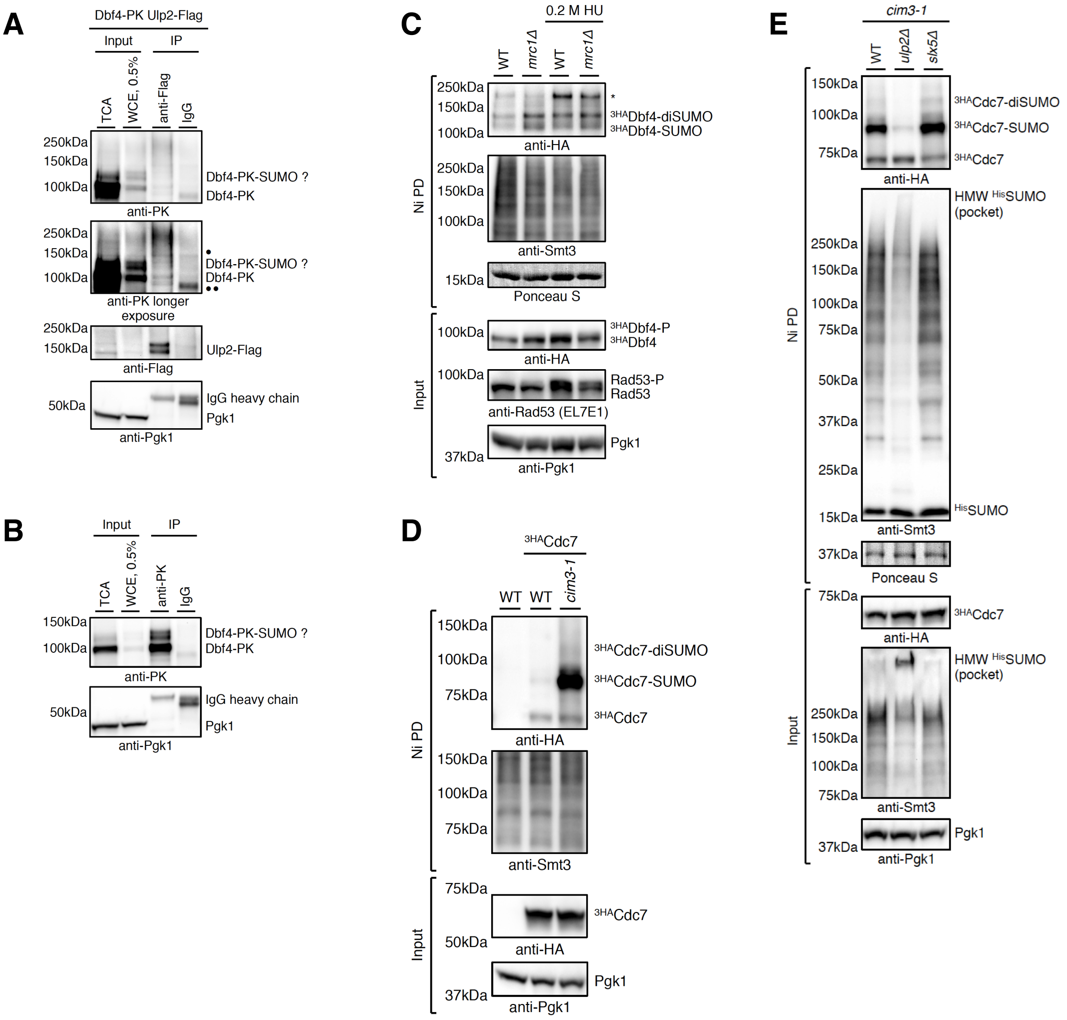

Figure S3. Chromatin-bound DDK Engaged in Replication is SUMOylated and Safeguarded by Ulp2 Against the Slx5/8 STUbL-mediated Proteasomal Degradation, Related to Figure 3.

(A) Ulp2 interacts with Dbf4 in the Co-IP studies with preference towards slower-migrating potentially SUMOylated Dbf4 species. Proteins were precipitated using trichloroacetic acid (TCA) to preserve the posttranslational modifications, or whole cell extracts (WCE) were prepared from cells expressing C-terminally 9PK-tagged Dbf4 and 3Flag-tagged Ulp2 using grinding in liquid nitrogen. Both unmodified and slower-migrating potentially SUMOylated species of Dbf4-PK Co-IP during IP with anti-Flag antibody, but not with mouse IgG. Single and double filled circles denote cross-reactivity of the anti-PK antibody.

(B) IP of slower-migrating potentially SUMOylated species of Dbf4-PK. Similar to (A), but Dbf4-PK and its modified species are immunoprecipitated specifically with anti-PK antibody and not with mouse IgG.

(C) Dbf4 SUMOylation is increased in the absence of S-phase checkpoint protein Mrc1. ^{His}SUMO Ni PD from untreated WT and *mrc1*Δ cells expressing ^{3HA}Dbf4 under the control of *ADH1* promoter, or grown to an OD_{600} of 0.7 and shifted to YPD containing 0.2 M HU for 90 min. Ni PD efficiency was assayed using anti-Smt3 antibody and staining with Ponceau S (nonspecifically-bound protein of ≈15 kDa visualized). Dbf4 phosphorylation (Dbf4-P) is lost and Rad53 phosphorylation (Rad53-P) is markedly reduced in *mrc1*Δ cells treated with HU compared to WT. Asterisk denotes cross-reactivity of the anti-HA antibody.

(D) SUMOylated Cdc7 species, but not unmodified Cdc7, strongly accumulate in the temperature sensitive *cim3-1* proteasome-defective mutant grown at permissive temperature (28°C) compared to WT cells. ^{His}SUMO Ni PD from WT and *cim3-1* cells expressing either untagged or N-terminally 3HA-tagged Cdc7 under the control of *ADH1* promoter. Unmodified forms of Cdc7 are nonspecifically pulled-down and can be detected in Ni PD.

(E) Turnover of degradation-prone monoSUMOylated Cdc7 is accelerated in the absence of Ulp2. Similar to (D), but with *cim3-1, cim3-1 ulp2*Δ*, and cim3-1 slx5*Δ cells grown at 28°C.

Figure S4

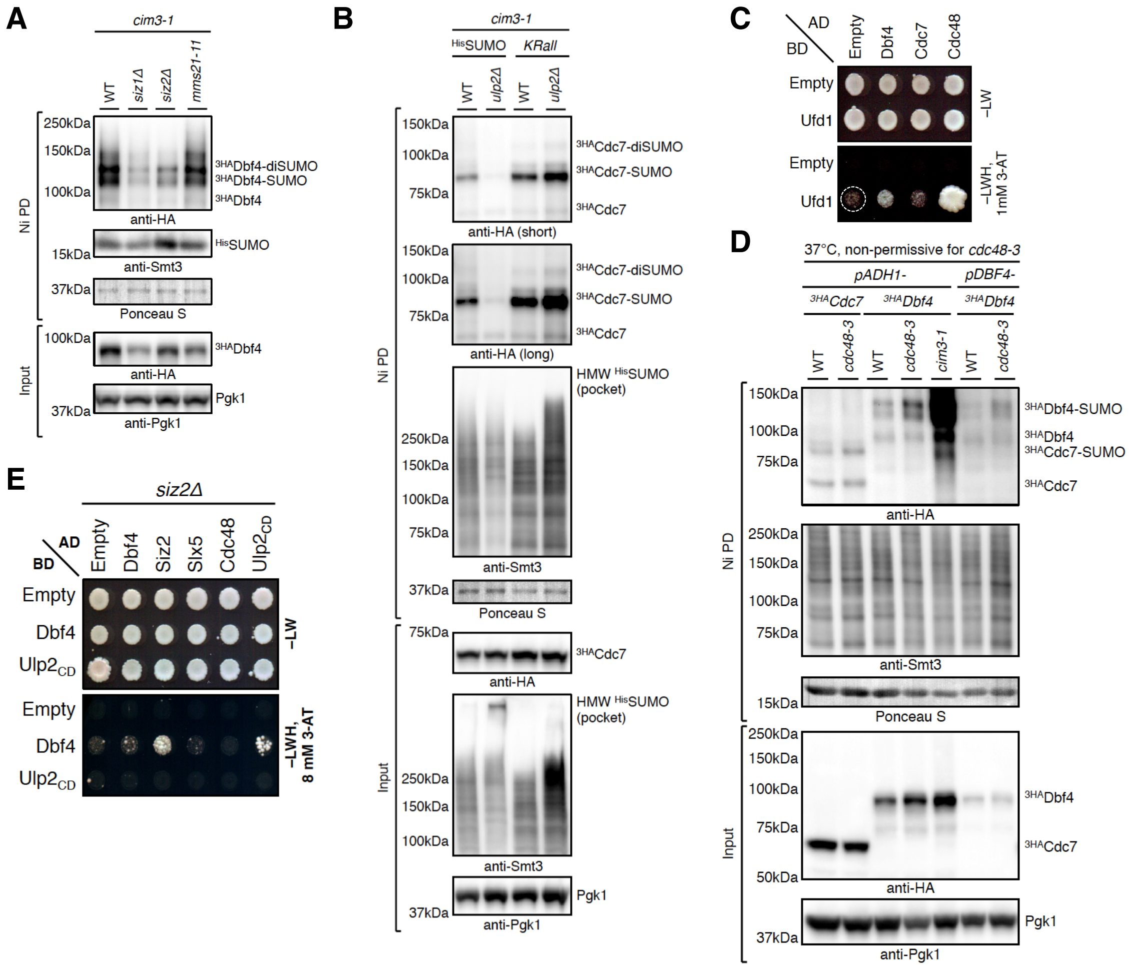

Figure S4. SUMO/Ubiquitin-dependent Segregase Cdc48 Assists in the Proteasomal Degradation of DDK Modified by SUMO Ligases Siz1 and Siz2, Related to Figure 4.

(A) SUMOylation of Dbf4 is mediated by the SUMO ligases Siz1 and Siz2. Ni PD of ^{His}SUMO conjugates from *cim3-1* mutant cells expressing ^{3HA}Dbf4 under the control of *ADH1* promoter (WT) and cells additionally lacking SUMO ligase Siz1 (*siz1*Δ), Siz2 (*siz2*Δ), or carrying *mms21-11* allele, a mutant of Mms21 that is deficient in SUMO ligase activity. Ni PD efficiency was assayed using anti-Smt3 antibody and staining with Ponceau S (nonspecifically pulled-down protein of ≈37 kDa visualized).

(B) The decreased levels of monoSUMOylated Cdc7 species in *cim3-1 ulp2*Δ mutant cells are restored if instead of ^{His}SUMO a lysine-less SUMO variant (*KRall*) that cannot form lysine-linked SUMO chains is expressed as the only source of SUMO.

(C) Dbf4 interacts with the Cdc48 segregase substrate-recruiting co-factor Ufd1 in the Y2H system. 1mM 3-amino-triazole (3-AT) added to reduce auto-activation of the *HIS3* reporter gene by BD-Ufd1 fusion alone (marked by circle).

(D) Cdc48 segregase assists in the proteasomal degradation of SUMOylated DDK. ^{His}SUMO Ni PD from WT cells and temperature sensitive *cdc48-3* mutant expressing ^{3HA}Cdc7 under the control of strong *ADH1* promoter (*pADH1*), and ^{3HA}Dbf4 either under the control of *ADH1* or endogenous promoter (*pDBF4*), grown to an OD_{600} of 0.7 at 28°C and then shifted to 37°C for 3 hours. SUMOylated DDK subunits accumulate in *cdc48-3* mutant compared to WT cells.

(E) Binding of Dbf4 to the Slx5 STUbL subunit is lost in the Y2H system, while interaction between Dbf4 and Ulp2 is strongly diminished, in the absence of the Siz2 SUMO ligase. Interaction between Ulp2 and Cdc48 is Siz2-dependent. 8mM 3-AT used to reduce background growth.

Figure S5

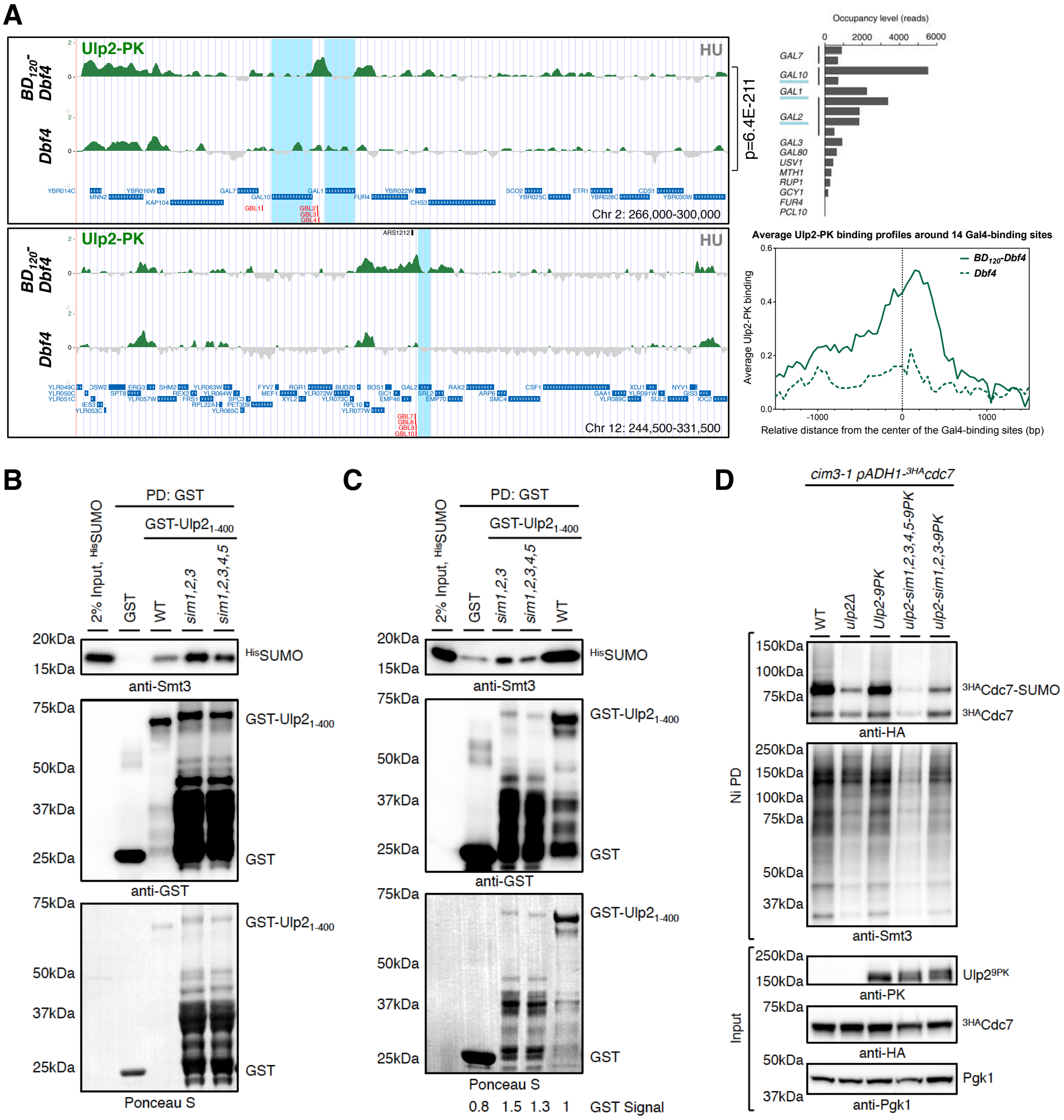

Figure S5. Ulp2 Is Recruited to Chromatin-bound SUMOylated DDK Artificially Targeted to Gal4-binding Sites and Uses Its N-terminal SIMs for Interaction, Related to Figure 5.

(A) N-terminal fusion of the Gal4 transcriptional activator DNA-binding domain to Dbf4 (BD_{120}-Dbf4) recruits Ulp2 to the Gal4-binding locations (GBL) at the genes of the yeast galactose regulon. ChIP-on-chip profiles of Ulp2-PK from cells expressing either N-terminally 3HA-tagged Dbf4 or BD_{120}-Dbf4 fusion under the control of *ADH1* promoter released into the S phase in the presence of 0.2 M HU for 90 min. Fragments of chromosomes 2 and 12 with the annotated GBLs in red and the

GAL genes with the highest Gal4 occupancy highlighted in blue are shown (left). The p value is related to the genome-wide overlap between the Ulp2-PK clusters in the two indicated strains. Average Ulp2-PK binding profiles in a window of 3 kbps centered at 14 GBLs (right).

(B-C) Recombinant GST-Ulp2$_{1\text{-}400}$ fusions with either first three (*sim1,2,3*) or all five (*sim1,2,3,4,5*) potential N-terminal SIMs mutated form multiple degradation products compared to WT GST-Ulp2$_{1\text{-}400}$ when expressed in *E. coli* that retain ability to pull-down free ^{His}SUMO, albeit with strongly reduced affinity (close to background in case of *sim1,2,3,4,5*).

(D) Both *ulp2-sim* mutants fail to protect monoSUMOylated Cdc7 against SUMO-chain/STUbL-mediated proteasomal degradation similar to *ulp2*Δ cells. ^{His}SUMO Ni PD from *cim3-1*, *cim3-1 ulp2*Δ, and *cim3-1* cells carrying either Ulp2-9PK or its SIM-mutant variants that express ^{3HA}Cdc7 under the control of *ADH1* promoter. monoSUMOylated Cdc7 species strongly accumulate in *cim3-1* and *cim3-1 Ulp2-9PK*, but not in *cim3-1 ulp2* double mutants. Unmodified Cdc7 is nonspecifically pulled-down and is detected below SUMO-modified species in Ni PD.

Figure S6

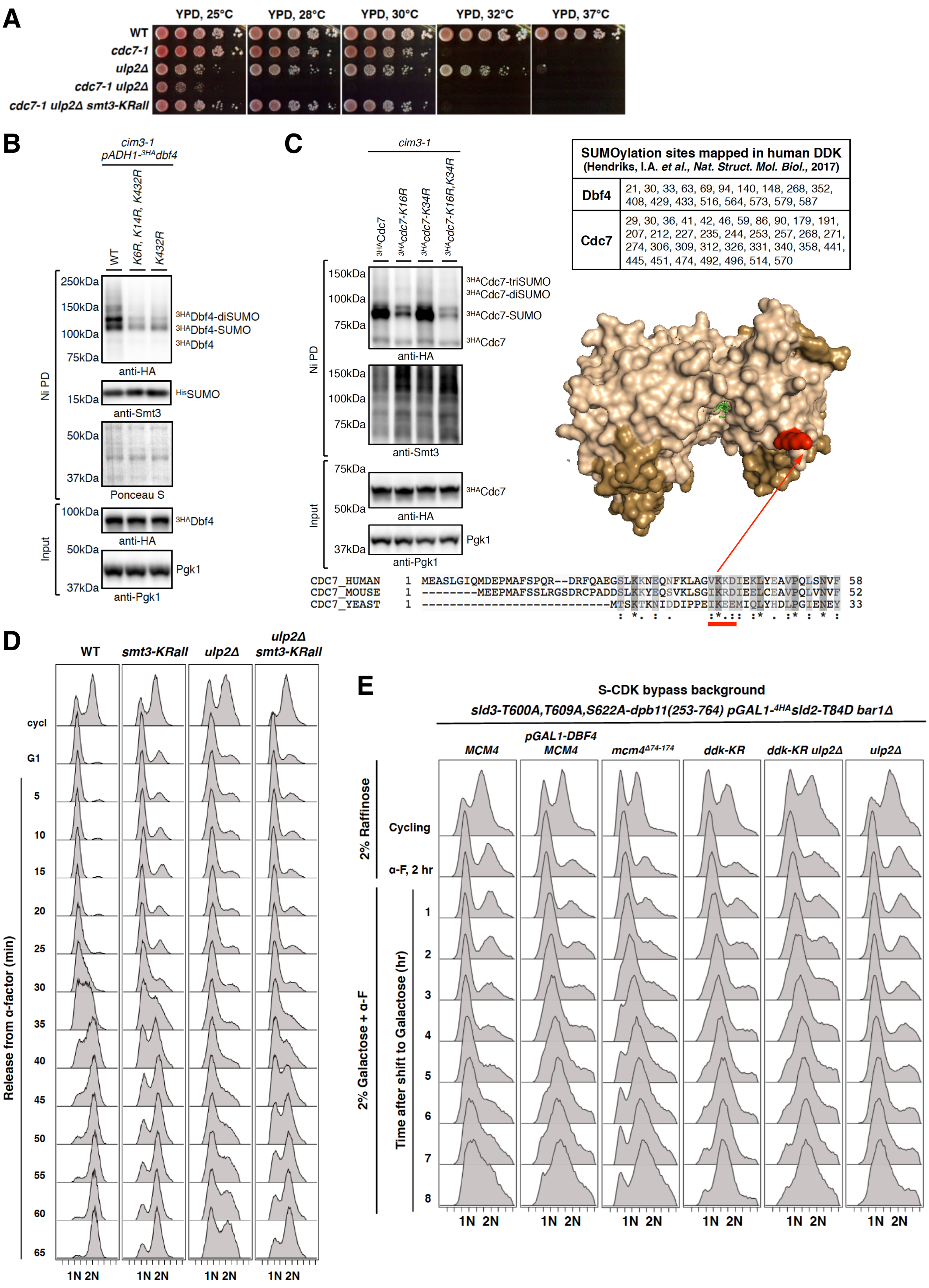

Figure S6. SUMOylation-defective *ddk-KR* Mutant No Longer Requires Ulp2 For Protection Against STUbL-mediated Proteasomal Degradation and Efficiently Promotes Replication Onset, Related to Figure 6.

(A) Temperature sensitive *cdc7-1* mutant exhibits synthetic lethal genetic interaction with *ulp2*Δ at permissive temperatures for *cdc7-1* cells that can be suppressed by *smt3-KRall* mutation. Spotting of 1:7 serial dilutions on YPD plates at indicated temperatures.

(B-C) Mapping of major SUMO acceptor sites in DDK and generation of a SUMOylation-defective *ddk-KR* mutant. Major SUMO acceptor lysines (K) were identified and replaced by arginines (R) to abolish the modification. ^{His}SUMO Ni PD from *cim3-1* proteasome-defective mutant cells expressing either N-terminally 3HA-tagged Dbf4 (B) or Cdc7 (C) and their various KR variants. Both DDK subunits are SUMOylated at multiple lysines, each contributing to a different extent to the overall DDK SUMOylation, as in the case of human DDK SUMOylation (C, above right). Cdc7 is largely SUMOylated at conserved K16. Structure of the human Cdc7-Dbf4 heterodimer (Hs_Cdc7, wheat; Hs_Dbf4, sand; C, below right) with ADP (green) modeled at active site. Validated SUMO acceptor lysine 16 (K16) in budding yeast Cdc7 lies within a SUMO consensus motif **ψ-K-x-E/D** (ψ, a hydrophobic amino acid; x, any amino acid), which is conserved in mouse and human (underlined red in the alignment of the Cdc7 N-termini from budding yeast, mouse and human), and corresponds to K41 in human Cdc7 (highlighted in red in the structure).

(D) S phase progression defect in *ulp2*Δ cells is suppressed by *smt3-KRall*. Exponentially growing WT, *smt3-KRall*, *ulp2*Δ, and *ulp2*Δ *smt3-KRall* cells (cycl) arrested in G1 phase by α-factor, released into YPD at 25°C, and samples taken every 5 minutes for FACS.

(E) Replication initiation defect in *ulp2*Δ S-CDK bypass background cells can be suppressed by the SUMOylation-defective *ddk-KR* mutant. Exponentially growing (Cycling) S-CDK bypass background cells (*MCM4*), *MCM4* cells harboring extra copy of *DBF4* under the control of galactose-inducible promoter (*pGAL1-DBF4*), *mcm4*$^{\Delta 74-174}$*, ddk-KR, ulp2*Δ*,* and *ulp2*Δ *ddk-KR* cells cultured in YP raffinose (2%) were arrested in G1 phase by α-factor (α-F), rapidly switched to YP galactose (2%) containing α-F at 28°C, and samples were taken every hour for FACS analysis.

Figure S7

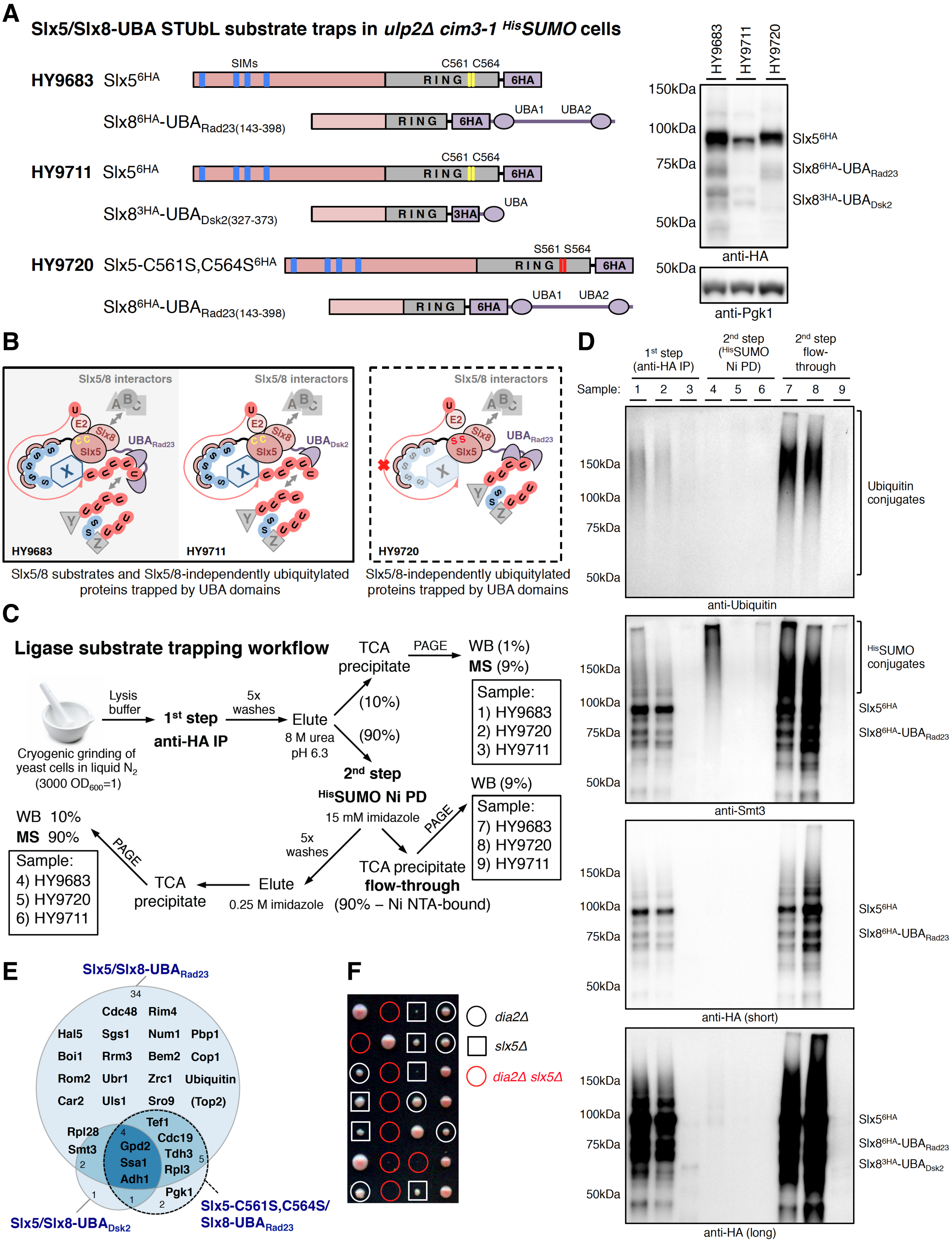

Figure S7. Slx5/8 STUbL Substrate Trapping in *cim3-1 ulp2Δ* ^{His}*SUMO* Background, Related to Figure 7.

(A) Schematic representation of the Slx5/Slx8-UBA substrate traps generated (strains HY9683, HY9711, HY9720) in *cim3-1 ulp2*Δ ^{His}*SUMO* background (left). UBA domains with different affinity towards ubiquitin chains derived either from Rad23 (aa 143-398; UBA1 and UBA2) or Dsk2 (aa 327-373) ubiquitin receptors were fused C-terminally to Slx8 via 6HA- or 3HA-containing linker sequences, respectively. Additionally, Slx5 was C-terminally tagged with 6HA in all strains with the only difference that in HY9720 point mutations (*slx5-C561S, C564S*) were introduced in the RING domain of Slx5 inactivating the Slx5/8 STUbL. Fusion of the Dsk2-derived UBA domain via 3HA to Slx8 destabilized the Slx5/8 STUbL (right).

(B) Schematic representation of the proteins predicted to bind to the generated substrate traps during the anti-HA IP. Catalytically active Slx5/Slx8-UBA traps may co-IP Slx5/8 interactors (labeled A, B, C), Slx5/8-independently ubiquitylated proteins (labeled Y, Z) and true Slx5/8 substrates (labeled X) in most cases marked with SUMO chains, whereas catalytically inactive slx5-C561S, C564S/Slx8-UBA fusion can only trap interactors and Slx5/8-independently ubiquitylated proteins, thus serving as a nonspecific background binding control.

(C) Schematic representation of the Slx5/8 ligase substrate trapping workflow and samples (1-9) taken for western blot (WB) procedure control (see panel D) or mass spectrometry (MS) analysis. Percentage in parentheses represents amount of immunoprecipitated material following 1st step anti-HA IP taken for subsequent steps: WB control, MS, 2nd step Ni-NTA pull-down (Ni PD) of ^{His}SUMO conjugates.

(D) Western blot control of the Slx5/8 ligase substrate trapping procedure. Both catalytically active and inactive stable Slx5/Slx8-UBA$_{Rad23}$ traps efficiently co-IP ubiquitin conjugates (samples 1, 2, 7, 8; anti-Ubiquitin), whereas SUMO conjugates – expected substrates of STUbL – are enriched after 2nd step ^{His}SUMO Ni PD only in strains expressing catalytically active Slx5 (samples 4 and 6; anti-Smt3), even despite of the low stability of Slx5/Slx8-UBA$_{Dsk2}$ trap (samples 3 and 9; anti-HA), but not when Slx5/8 STUbL is inactive (sample 5; anti-Smt3).

(E) Euler diagram representing proteins detected by mass spectrometry following 2nd step ^{His}SUMO Ni PD of the Slx5/8 STUbL substrate trapping procedure (samples 4, 5, 6 in panels C and D). Proteins (see also Table S1) were identified using Scaffold software with stringent criteria; Top2 (in parentheses) was detected with one unique peptide.

(F) Mutations of the F-box protein Dia2 that drives MCM helicase ubiquitylation during DNA replication termination (*dia2*Δ) and of the Slx5/8 STUbL (*slx5*Δ) are synthetically lethal. Tetrad dissection of the *DIA2*/*dia2*Δ *SLX5*/*slx5*Δ diploid yeast.
